# CYP6P9-Driven Signatures of Selective Sweep of Metabolic Resistance to Pyrethroids in the Malaria Vector *Anopheles funestus* Reveal Contemporary Barriers to Gene Flow

**DOI:** 10.3390/genes11111314

**Published:** 2020-11-05

**Authors:** Delia Doreen Djuicy, Jack Hearn, Magellan Tchouakui, Murielle J. Wondji, Helen Irving, Fredros O. Okumu, Charles S. Wondji

**Affiliations:** 1 LSTM Research Unit, Centre for Research in Infectious Diseases (CRID), P.O. Box 13591 Yaoundé, Cameroon; magellan.tchouakui@crid-cam.net (M.T.); murielle.wondji@lstmed.ac.uk (M.J.W.); 2 Department of Vector Biology, Liverpool School of Tropical Medicine, Pembroke Place, Liverpool L3 5QA, UK; jack.hearn@lstmed.ac.uk (J.H.); helen.irving@lstmed.ac.uk (H.I.); 3 Environmental Health and Ecological Sciences Department, Ifakara Health Institute, PO Box 53 Ifakara 67501, Tanzania; fredros@ihi.or.tz

**Keywords:** resistance, *Anopheles funestus*, *CYP6P9a* gene, gene flow, Africa

## Abstract

Pyrethroid resistance in major malaria vectors such as *Anopheles funestus* threatens malaria control efforts in Africa. Cytochrome P450-mediated metabolic resistance is best understood for *CYP6P9* genes in southern Africa in *An. funestus*. However, we do not know if this resistance mechanism is spreading across Africa and how it relates to broader patterns of gene flow across the continent. Nucleotide diversity of the *CYP6P9a* gene and the diversity pattern of five gene fragments spanning a region of 120 kb around the *CYP6P9a* gene were surveyed in mosquitoes from southern, eastern and central Africa. These analyses revealed that a *Cyp6P9a* resistance-associated allele has swept through southern and eastern Africa and is now fixed in these regions. A similar diversity profile was observed when analysing genomic regions located 34 kb upstream to 86 kb downstream of the *CYP6P9a* locus, concordant with a selective sweep throughout the rp1 locus. We identify reduced gene flow between southern/eastern Africa and central Africa, which we hypothesise is due to the Great Rift Valley. These potential barriers to gene flow are likely to prevent or slow the spread of *CYP6P9*-based resistance mechanism to other parts of Africa and would to be considered in future vector control interventions such as gene drive.

## 1. Introduction

*Anopheles funestus* is one of the most important malaria vectors in Africa. The vector has a wide geographic distribution ranging from south of the Sahara Desert to northern South Africa and Madagascar [1]. In many of these areas, *An. funestus* is the dominant malaria vector, due to its highly anthropophilic and endophilic preferences [2,3]. Sub-Saharan Africa where more than 90% of the world-wide burden of malaria occurs, has witnessed unprecedented progress in malaria prevention and control in the last two decades, with around a 70% reduction in malaria mortality [4]. This has mainly been the result of a significant scaling up of vector-control interventions, relying extensively on the use of insecticide-treated nets and residual spray inside houses [5,6,7]. Unfortunately, the propensity of *Anopheles* mosquitoes to develop resistance to various types of insecticides is endangering these fragile gains [8]. Resistance to pyrethroid insecticides is of particular concern because they are the main class of insecticide, with low toxicity to humans, that are currently recommended for impregnation into bed nets [9]. This major challenge for control tools, highlights the urgent needs for a better understanding of resistance evolution by assessing the speed and spread of resistance in major malaria vectors.

Resistance to pyrethroids has been reported in many populations of malaria vectors in the southern part of Africa, notably in *An. funestus* [10,11,12,13,14,15]. Investigations of underlying resistance mechanisms revealed that cytochrome P450-based metabolic resistance is the main mechanism involved in the observed resistance [16,17]. Moreover, selective sweeps have been reported to be associated with this metabolic resistance, driven by the scale-up of insecticide-based malaria control [18,19]. In the context of insecticide resistance escalation [20], continuous monitoring of spread of resistance and selection levels is necessary to predict control failure and recommend alternative insecticides. The tandemly duplicated *An. funestus* cytochrome P450s *CYP6P9a* and *CYP6P9b* genes were identified as the main drivers of resistance in Mozambique and southern Malawi [16,17,19], yet it remains unclear whether the confinement of this mechanism to the southern part of Africa is due to unidentified phylogeographic barriers to its spread, or insufficient time for continent-wide dispersion to occur. Significant genetic structure has been previously revealed among *An. funestus* populations across Africa [18,19,20,21] in line with pre-existing data [22]. It remains to be established how such population structure impacts the spread of P450-based resistance, which is the predominant resistance mechanism in *An. funestus*. Monitoring of the genetic diversity and signatures of selection on P450-based resistance loci, such as that driven by *CYP6P9a*, is necessary to better understand the dynamic and evolution of such major mechanism of resistance to insecticides in malaria vectors.

Here, we provide an overview of the genetic diversity and selection acting on the major pyrethroid resistance gene *CYP6P9a* across southern and East Africa and infer the dynamics of the spread of this resistance allele between the different mosquito populations. We confirm the presence of a strong selective sweep spanning a 120 kb previously-identified major cytochrome P450-based pyrethroid resistance locus [17,18] with a contrasting signature between regions highlighting the likelihood of previously reported barriers to gene flow in this species.

## 2. Materials and Methods

### 2.1. Sampling

**Figure 1:**
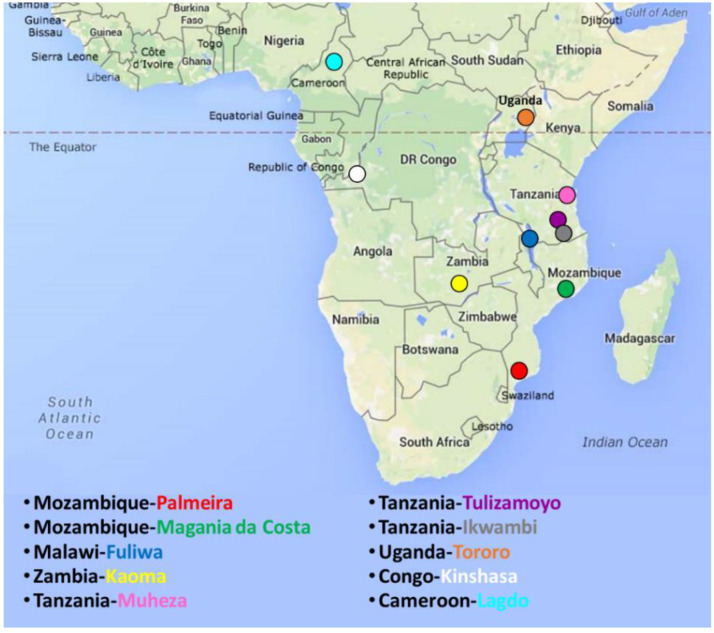
Map of Sub-Saharan Africa showing the sampling locations. Circle colour corresponds to sampling site colouring in the legend.

*Anopheles funestus* mosquitoes were collected between 2012 and 2018 from nine localities in six countries in southern, eastern and central Africa (Figure 1). The sampling locations were: Palmeira [20] and Magania-da-Costa in Mozambique (MOZ), Fulirwa in Malawi (MAL), Kaoma [21] in Zambia (ZB), Muheza, Tulizamoyo and Ikwambi in Tanzania (TZ) [23], Tororo [24] in Uganda (UG) and Kinshasa [25] in Democratic Republic of Congo (DRC). Further details are presented in (Table S1). Additional analyses were performed using sequencing data accessions of mosquitoes from Lagdo in Cameroon (CAM) reported in a previous study (Accessions KU168978 to KU168995) [18]. All mosquitoes were dissected into “ead and thorax” and “Abdomen” samples prior to DNA extraction. Genomic DNA was extracted from each part of the mosquito using the Livak method [26] or the DNeasy DNA extraction Kit (Qiagen Inc., Valencia, CA, USA). Head and Thorax samples were molecularly identified as *An. funestus* using a cocktail PCR [27] and further confirmed where ambiguous with primers targeting the internal transcribed spacer region 2 (ITS2) of ribosomal DNA as previously described [27]. Only confirmed *An. funestus sensu stricto* (hereinafter referred to as *An. funestus*) mosquitoes were used in this study.

### 2.2. PCR Amplification and Sequencing of CYP6P9a Gene and rp1 Fragments

Nucleotide diversity in the *CYP6P9a* gene and the surrounding genomic region was assessed by PCR amplicon sequencing. Primers from Riveron et al. [16] were used to amplify a portion of the 5′ upstream intergenic region, the untranslated region, 1470 bp coding sequence of the two exons and the gene’s unique intron for a total length of ~2.2 kb [17]. Five additional loci spanning the 120 kb BAC region located in the *resistance-to-pyrethroids 1* (rp1) quantitative trait loci (QTL) region of the 2R chromosome of *An. funestus* genome were also amplified in order to assess the presence of a selective sweep around *CYP6P9a* across Africa. These loci: 0 BAC, 25 BAC, 63 BAC, 95 BAC and 120 BAC were originally characterized in the laboratory resistant strain, FUMOZ-R, and are located at −34 kb, −9 kb, +29 kb, −61 kb and +86 kb upstream and downstream of the start of the *CYP6P9a* locus respectively [17,18]. For each *rp1* fragment located upstream and downstream of *CYP6P9a*, one pair of primers previously published [18] was used to amplify fragments of genomic DNA approximately 700 bp long (Figure S1, Table S2).

PCRs were performed by 15 μL amplification reaction consisting of 0.02 unit of Phusion Hot Start II High-Fidelity DNA Polymerase (Thermo Fisher Scientific, Waltham, MA, USA) for *CYP6P9a* and 1 unit of KapaTaq (Kapa Biosystems, Wilmington, MA, USA) for BAC clone. Reactions were added of 0.2 mM dNTPs, 0.5 μM of each primer, respective buffer solutions for GC rich templates and at least 10 ng of genomic DNA (1 µl). PCR reactions were carried out in PCR System 2720 thermal cycler (Applied Biosystems, Foster City, CA, USA) under the following conditions for *CYP6P9a* gene: 1 min at 98 °C, followed by 35 cycles of 10 s at 98 °C, 30 s at 62 °C and 2 min at 72 °C and a final extension of 10 min at 72 °C. BAC fragments was amplified using a program of 5 min at 95 °C, followed by 35 cycles of 30 s at 95 °C, 30 s at a primer-specific temperature (Table S2), and 1 min at 72 °C and, for a final extension that lasted 10 min at 72°C. 

PCR products were purified using the EXOrSAP reagent (New England Biolabs, Ipswich, MA, USA) or the QIAquick PCR Purification Kit (Qiagen) and directly sent for sequencing using PCR primers. We designed additional internal overlapping primers (Table S2) for sequencing the *CYP6P9a* locus, in order to ensure coverage of the full-length sequence. To ensure reliability of the sequencing data, PCR amplicons were sequenced in both directions, and any remaining ambiguities in the sequences resolved by repeated PCR amplification and re-sequencing.

### 2.3. Data Analysis

Sequence alignment was conducted with the ClustalW module of MEGA X version 10.1 [28]. Heterozygous sites in sequence data were phased using the PHASER algorithm implemented in DnaSP version 6.12.01 (University of Barcelona, Barcelona, Spain) [29]. DnaSP was also used to identify the number of segregating sites (S), levels of nucleotide diversity Pi (π), the average number of nucleotide differences per site between any two sequence, and for testing deviations from neutrality at each locus through Tajima’s D, Fu and Li’s D* and Fu’s FS indexes.

Haplotype analysis was conducted using DnaSP to count the number of haplotypes (h) and the haplotype diversity (Hd) of each locus. TCS software [30] and its constituent program tcsBU beautifier [31] were used to generate haplotype networks based on a 95% connection limit with gaps treated as a fifth state. Haplotypes were labelled by colour and shape where the size of the shapes are proportional to the frequency of the haplotype and space between circles on each branch show the mutational steps separating haplotypes.

Phylogenetic trees generated by the Maximum likelihood (‘ML’) and Neighbour joining (NJ) methods were inferred on *CYP6P9a* and BAC sequences using MEGA X. Before running ML trees, final alignments were submitted to MEGA X Models program to select the best model for phylogenetic analysis based on Bayesian and Akaike Information Criterions (BIC and AICc). The evolutionary history of all sequences was derived using the Maximum likelihood method based on the Kimura 2-parameter (K2) or the Hasegawa-Kishino-Yano with γ distribution (HKY+G) models. The tree with the highest log likelihood was selected. To test the robustness of tree topologies, 1000 bootstrap replicates were performed. Trees were drawn to scale, with branch lengths measured in the number of substitutions per site. For *F_ST_* distance pairwise comparisons, Neighbor-Joining trees were generated and optimal trees with bests sum of branch length were selected.

For protein coding sequences, 642 bp of the *CYP6P9a* gene and 750 bp of the 25 BAC fragment, we calculated piN/piS ratios (non-synonymous polymorphism rate/polymorphism substitution rate) between the different populations to investigate selection pressure on the gene. A piN/piS ratio significantly greater than one implies positive selection, less than one implies purifying selection, and a ratio of one indicates a selectively neutral locus.

We estimated genetic diversity in the different geographical populations by calculating population differentiation indexes in DnaSP software. *F_ST_*_,_ the fixation index shows the overall differentiation by measuring how likely, two homologous genes, randomly chosen in a subpopulation, result from a single gene in the subpopulation [32,33,34]. *G_ST_* although analogous to the previous, was calculated to double check the *F_ST_* values and verify the consistency of the results. *G_ST_*, is a multi-allele generalization of *F_ST_*, which was defined as the relative difference between the expected heterozygosity of the whole population and the mean expected heterozygosity of the individual demes [33,35]. The *K_ST_* statistic was used to estimate the levels of pair-wise genetic differentiation between populations in terms of the relative number of shared alleles among the populations, without regard to their frequencies [33,36]. *H_ST_* which is related to haplotype frequency was also calculated. *H_ST_* is a differentiation metric which weighs alleles by their population frequency [33]. High *H_ST_* values increase with the addition of private alleles in a population while it will decrease when a given allele dominating in the population will rise in frequency. Finally, *N_ST_* has been calculated to take into account the phylogenetic relationships between haplotypes, and to test the occurrence of a phylogeographic pattern in the dataset [33,34] as an *N_ST_* significantly greater than *G_ST_*, implies a phylogeographic structure [37]. *N_ST_* estimates were associated with the number of migrations per generation (Nm) to help evaluate gene flow among studied populations. The statistical significance of *H_ST_* and *K_ST_* estimates was assessed by permutation of subpopulations identities and recalculating indexes 10,000 times as implemented in DnaSP 6.12.

## 3. Results

### 3.1. Sequence Diversity at CYP6P9a

Sequences were first examined across the entire 2.2 kb length of the *CYP6P9a* locus. After alignment and assessment in MEGA X [28], a consensus sequence of 1139 bp length was obtained for 111 *An. funestus* field-collected mosquitoes from ten localities in seven countries (Mozambique (Palmeira and Magania-da-Costa), Malawi (Fulirwa), Zambia (Kaoma), Tanzania (Muheza, Tulizamoyo and Ikwambi), Uganda (Tororo), Democratic Republic of Congo (DR Congo, Kinshasa) and Cameroon (Lagdo)) for which good sequence data were obtained. This included 465 bp of the partial 5′ upstream intergenic region, 32 bp of 5′UTR and 642 bp of partial gene’s first exon. The examination yielded 84 single nucleotide polymorphisms (SNPs) and 6 insertions or deletions (Indels) in the 1.139 bp region (Figure S2), of which 49 polymorphism sites (47 SNPs and 2 indels) were observed in Cameroon and DR Congo and most were located in the non-coding region. Again, Southern African mosquito populations and subpopulations displayed lowest polymorphic sites (0 to 4 in the coding region) while the highest one was observed in Cameroon (16 segregating sites in the coding region) (Table 1).

All sites in Mozambique presented similar patterns of low diversity with respectively 0.00009 and 0.00016 nucleotide diversity (π) found in Palmeira and Magania da Costa. Similarly, all sites in Tanzania presented similar patterns of low diversity with π ranging from 0.00010 to 0.00033. Overall, nucleotide diversity was lowest in both countries, Mozambique (0.00014) and Tanzania (0.00027), compared to Zambia (0.00042), Malawi (0.00061) and Uganda (0.00109). Cameroon and DR Congo displayed higher but comparable diversity (respectively 0.00200 and 0.00304) corresponding to their geographical proximity (Table 1). Nucleotide diversity was highest in central African countries. These results indicate that the diversity of *CYP6P9a* gene was lower in the Southern and Eastern African countries surveyed, than in Central African. We observed the same pattern of nucleotide diversity when focusing only on the coding and non-coding region of the analysed fragment, with the exception of Uganda. Indeed, in all Southern African countries, the diversity remains very low compare to central African one. In Uganda however, no polymorphism is noted in the coding region, while in the non-coding region, the observed diversity is even higher than in Cameroon (Table 1).

**Figure 2:**
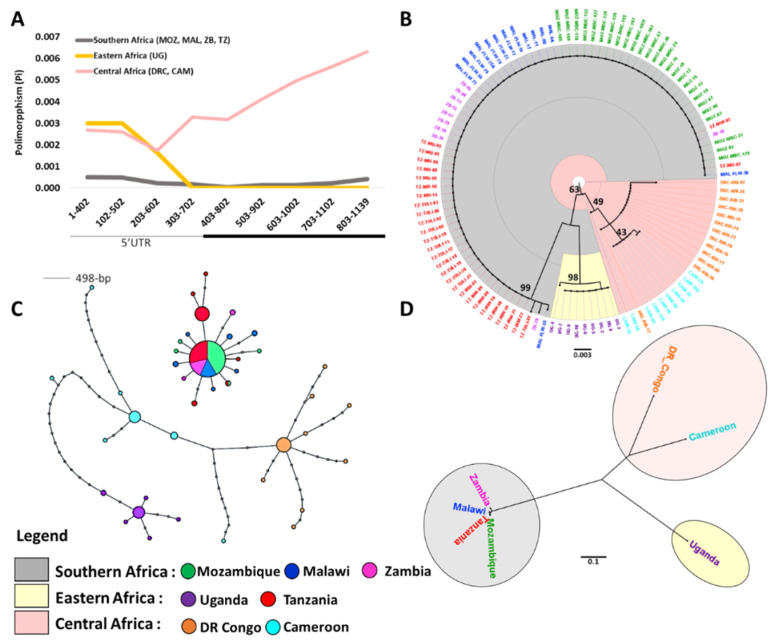
Analysis of genetic diversity in *CYP6P9a* key pyrethroid resistance gene in *An. funestus* populations across seven different African countries. (**A**) Sliding-window analysis of nucleotide polymorphism (Pi) in Southern Africa (158 accessions), Eastern (46 accession) and Central Africa (18 accessions). Pi was calculated for segments of 400 bp at 100 bp intervals. The black bar shows *CYP6P9a* gene’s exon. (**B**) Maximum likelihood tree of the *CYP6P9a* gene sampled from throughout Africa, showing clear geographical divergence between southern Africa (MZ, MAL, ZB and TZ; 99% bootstrap support), East Africa (UG; 98% bootstrap support) and Central Africa (DRC and CAM; 49% bootstrap support). (**C**) Haplotype network for non-synonymous nucleotide variants in CYP6P9a. Mosquito populations from MOZ, MAL, ZB and TZ form a defined clade from UG, DRC and CAM populations, and are dominated by a single Major haplotype. UG forms a distinct haplotype related to CAM and DRC populations that also form two distinct but related haplotypes. Haplotypes are labelled by colour and shape where the area of each circle is proportional to the frequency of the haplotype it represents. Lines connecting haplotypes and each node represent a single mutation. (**D**) Phylogenetic tree generated by the Neighbor-Joining method based on pairwise F_st_ distance obtained from 1139-bp *CYP6P9a* gene, including 111 *A. funestus* sequences from seven African countries according to each sample’s country of origin.

Results of Tajima’s D tests were significantly negative in all mosquito populations and sub populations, showing a deviation from neutrality (Table 1). This result is confirmed in both coding and non-coding regions where, instead of having non-computed D—because of the absence of polymorphism in the assessed population—Tajima’s D tests were also negative. Significant and negative Fu and Li’s D* and Fu FS values are concordant with positive selection indicating a strong selective sweep at this locus (Table 1). Sliding window analysis of the pattern of polymorphism across *CYP6P9a* gene reveal a loss in diversity in the entire first exon of the gene in southern and eastern African mosquito populations as highlighted by the sharp peak at around 400 bp in sequences from central Africa (Figure 2a). These results also suggest strong selection at *CYP6P9a*, specifically in the coding region of the gene. Notably, only few SNPs (7 of 41) in the first exon coding region of *CYP6P9a* that occurred in Mozambique, Malawi, Zambia and Tanzania were non-synonymous (Figure S2).

To identify if this result is due to positive selection, pN/pS ratios were calculated between CYP6P9a non-synonymous and synonymous site among the different countries. Ratios were much greater than 1.0 in DR Congo (1.62) suggesting strong positive selection of CYP6P9a resistance allele in the country. In mosquito populations from Zambia and Mozambique however, purifying selection was observed as shown by ratios values that were less than one (0.31 to 0.94 respectively). In other countries where there is no synonymous and/or non-synonymous substitution, we were not able to calculate the ratio (Table 2).

### 3.2. Maximum Likelihood Phylogenetic Tree and Haplotype Network for CYP6P9a

Phylogenetic analysis further confirms the selection acting on *CYP6P9a* in southern Africa and Tanzania where less diversity and divergence is observed, but not in other parts of the continent where we had higher divergence among individuals of the same population (Figure 2b, Figure S3). The maximum likelihood tree of the *CYP6P9a* full length fragment, as well as those based on non-synonymous SNPs, coding and non-coding regions of the gene sampled throughout Africa, shows a clear geographical divergence between mosquito populations from Southern Africa (MOZ, MAL, ZB) along with Tanzania (TZ), Eastern (UG) and Central Africa (CAM, DRC). Mosquito populations from Southern Africa plus Tanzania share the same cluster, while those from Uganda and Central Africa form two distinct clades driven by the same branch (Figure 2b, Figure S3). Analysis of the TCS haplotype network of *CYP6P9a* confirms profiles obtained with the ML tree. *CYP6P9a* from MOZ, MAL, ZB and TZ form a defined clade from UG, CAM and DRC populations, dominated by a single major haplotype (frequency of 118). UG forms a distinct haplotype related to CAM and DRC also forming two distinct but related haplotypes with respect to their geographical proximity (Figure 2c). When considering only Non-synonymous mutations, the trend is the same with however UG, CAM and DRC forming three unrelated haplotypes (Figure S4).

### 3.3. Genetic Differentiation of the CYP6P9a Gene

Genetic differentiation among populations was tested using *F_ST_* values, which show the overall fixation index and *N_ST_* values that show phylogeographic relation between haplotypes. An *Nm* value associated with *F_ST_*, reflecting the number of migrations, was also calculated to evaluate gene flow among studied populations. *F_ST_* values showed a very low genetic differentiation between southern African and Tanzanian mosquito populations (*F_ST_* = 0.02) and an associated high gene flow between them (*Nm* = 10.87). There were higher levels of genetic differentiation among populations from Central Africa and Uganda than when compared to those from southern Africa (*F_ST_* = 0.53 to 0.97) (Tables S3 and S4). The same trend is observed when considering only coding or non-coding regions of analysed sequences (Tables S3 and S4). These results were consistent with *G_ST_*, *K_ST_* and *K_ST_* estimates, as well as the Neighbour-Joining tree based on *F_ST_* distance values (Figure 2d; Tables S3 and S4). Moreover, *N_ST_* values were significantly greater than *G_ST_* values when comparing central, eastern and southern African mosquito population each other. The same observation was made for populations from the two central African countries (Cameroon and DR_Congo) but not for southern African populations (Table S3). This result implies there is a phylogeographic structure between compared haplotypes except those from southern Africa.

### 3.4. Selective Sweep Surrounding CYP6P9a

To assess the impact of selection at *CYP6P9a*, the extent of nucleotide polymorphism was examined at five loci around the resistance gene, within the BAC genomic region spanning the rp1 QTL, approximately 120 kb long (Figure S1). For better assessment of the nucleotide diversity, results were further summarized by grouping samples into southern (MOZ, MAL, ZB, TZ), Eastern (UG) and Central (DRC, CAM) Africa. Taking into account the phylogenetic and diversity results, Tanzania was now considered along with southern African samples and Uganda was the only representative of eastern Africa diversity.

**Figure 3:**
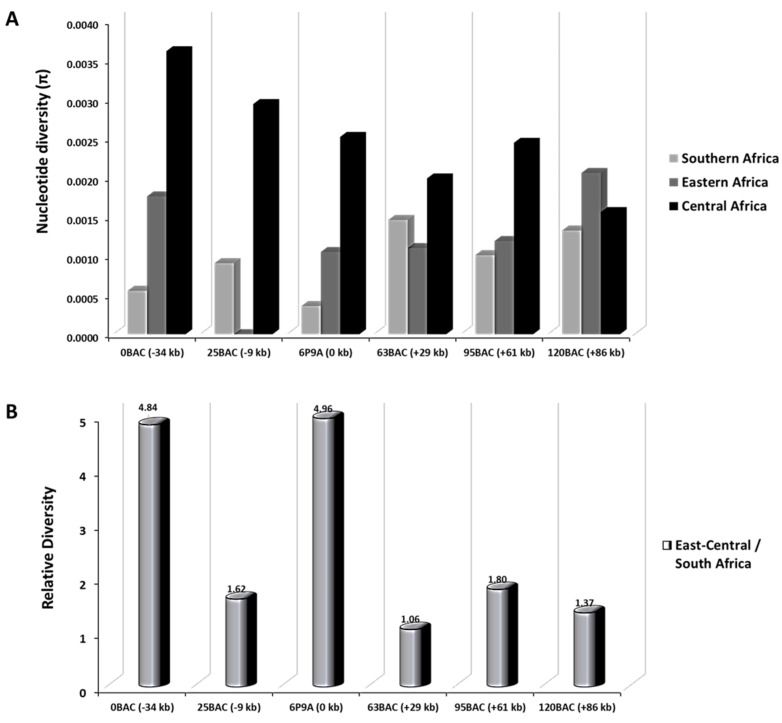
Nucleotide diversity survey at *CYP6P9a* and five loci around the pyrethroid resistance gene. For each locus, nucleotide diversity was investigated by sequencing *A. funestus* samples from seven sub-Saharan African countries. Results were further grouped samples into Southern, Eastern and Central Africa respective to mosquito field population origin. Positions (kb) of sequenced regions relative to CYP6P9a. (**A**) Levels of nucleotide diversity (π) for each African region. (**B**) Ratio of nucleotide diversity at each locus between Eastern-Central and Southern Africa.

The average nucleotide diversity (π) for southern African (plus Tanzanian) mosquito populations was lower at all six loci including *CYP6P9a* gene locus compare to that of populations from central Africa (Figure 3a and Figure S5). In Eastern Africa, absence of polymorphic sites was noted at the 25 BAC (−9 kb) locus while higher nucleotide diversities were observed at loci further away: 0.0018, 0.0011, 0.0012 and 0.0020 for loci 0BAC (−34 kb), 63BAC (+29 kb), 95BAC (+61 kb) and 120 BAC (+86 kb) respectively (Figure 3a, Table 3). Analysis of mosquito populations from DR Congo and Cameroon, revealed higher levels of polymorphism (ranging in average from 0.0016 to 0.0036 for all analyzed loci) across the *rp1* genomic fragment (Figure 3a, Table 3). This was principally the case in Cameroon with highest observed polymorphism levels in this study, ranging from 0.0030 to 0.0065 genetic diversity (Table 3, Figure S5). The relative levels of nucleotide diversity between eastern-central and southern mosquito populations were also calculated across the *rp1* genomic region (Figure 3b, Figures S5 and S6a,b, Table S5).

A striking reduction in the relative nucleotide diversity is observed between Eastern/Central and southern Africa (ratio ranging from 1.1 to 1.8) at loci from −9 kb to +86 kb, but markedly high at−34 kb and CYP6P9a loci (4.84 and 4.96 respectively) (Figure 3b, Table 3 and Table S5). Overall, a considerable change was seen from −34 kb to +86 kb (0 BAC to 120 BAC) around the *CYP6P9a* and *CYP6P9b* loci as well as other P450s and esterase loci (Table S6) where a strong signature of positive directional selection was observed in southern and to a lesser extend in eastern Africa. Taken together, these results support a selective sweep having occurred around *CYP6P9a* resistance gene, extending at least 100 kb in length in southern Africa.

Similar patterns were drawn from haplotype diversity measures. Average Hd values were lower for southern African mosquito populations for four loci along from −34 kb to +86 kb (but not at locus +29 kb) than for the *CYP6P9a* resistance gene locus. The same is true for eastern Africa. However, in central Africa average Hd values were much higher across all six loci including the gene *CYP6P9a* (Table 3). Tajima’s D tests were most often negatively significant, a signature of selection or population expansion, for the *CYP6P9a* locus in southern and Eastern African countries, however at least one locus has a significant Tajima’s D in each sampled location (Table 3).

**Figure 4:**
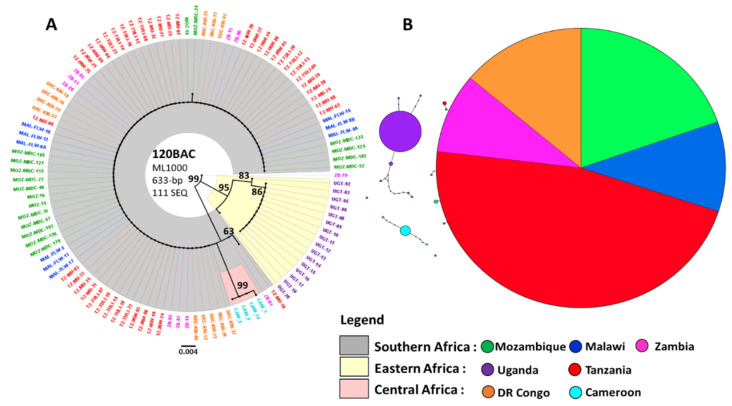
Analysis of genetic diversity across the rp1 pyrethroid resistance locus, example of the 120 BAC locus (**A**) Maximum Likelihood phylogenetic tree generated using the Hasegawa-Kishino Yano plus γ distribution model inferred on a 633-bp fragment of the 120 BAC locus of 111 *A. funestus* sequences from 7 sub-Saharan African countries. Each split is labelled with its bootstrap support value from 1000 bootstrap replicates. The tree is drawn to scale, with branch lengths measured in the number of substitutions per site. (**B**) Haplotype network for nucleotide variants in 120BAC fragments sequences showing that DR Congo mosquitos cluster with southern African versus mosquitos from Uganda and Cameroon.

Phylogenetic analyses of the five BAC fragment sequences from mosquito populations sampled throughout Africa highlighted a reduced diversity across the entire 120 kb portion of the *rp1* QTL studied. Construction of maximum likelihood (“ML”) phylogenetic trees revealed reduced diversity close to the *CYP6P9a* locus in the resistant populations of Mozambique, Malawi, Zambia and Tanzania. Uganda and DR Congo plus Cameroon samples show different profiles, forming distinct clusters (Figure 4a and Figure S7). The ML tree of the portions located −34 kb (0 BAC) and −9 kb (25 BAC) upstream the CYP6P9a gene, as well as +29 kb (63 BAC) and +61 kb (95 BAC) downstream, show that all the sequences from southern Africa (MOZ, MAL, ZB and TZ) are highly similar and belong to a unique major haplotype while those from UG and CAM plus DRC forms two distinct haplotypes (Figure S7a–d). The profile is a bit different +86 kb (120 BAC) far from the gene, 120 BAC sequence analysis reveals a profile which is typically the same as the one of the loci −34 kb. The only difference is the additional sequences from DRC mosquito populations that now cluster with the major haplotype (Figure 4a). The phylogeny of the BAC fragments sampled throughout Africa show a clear geographical divergence between southern Africa (MOZ, MAL, ZB and TZ) and eastern/central Africa (UG, DRC and CAM): mosquito populations from southern Africa share the same cluster, while those from eastern and central Africa form two distinct clades.

As for CYP6P9a, haplotype networks of the five up- and down-stream loci confirmed profiles obtained with the ML tree. In general, Mozambique, Malawi, Zambia and Tanzania formed their own cluster, with reduced genetic diversity shown by the fact that haplotypes from each country had no mutational steps between them. Samples from Uganda and DR Congo plus Cameroon formed their own clusters with few mutational steps between them for almost all of the BACs except for +61 kb (95 BAC) and +86 kb (120 BAC) loci (Figure S8). The significant difference in the 95 BAC loci is the small cluster (deriving from the major haplotype) formed by eleven sequences from Mozambique, Zambia and Tanzania (Figure S8d). In the 120 BAC loci, the only change is the fact that DR Congo sequences now cluster with the major haplotype (Figure 4b). From −34 kb to +61 kb (0 to 95 BAC) around *CYP6P9a* a strong signature of positive directional selection was observed in southern as well as in eastern but not in central Africa. This reduced haplotype diversity is shown by the presence of a highly predominant haplotype MAL/MOZ/ZB/TZ only in southern and part of eastern Africa (Figure 4b). 

Genetic differentiations parameters revealed patterns that were similar to those of the resistance gene *CYP6P9a*. A very low genetic differentiation associated with a high geneflow was observed between southern African mosquito population. Populations from central and eastern Africa exhibited lower levels of genetic differentiation and gene flow between them compared to those from southern Africa (Figure S9, Tables S7 and S8).

## 4. Discussion

### 4.1. Genetic Diversity and Selection on CYP6P9a

Insecticide resistance among malaria vectors is expanding across Africa, threatening the success of malaria control methods. Continuous monitoring of the evolutionary patterns of vectors population behaviour regarding insecticide-based interventions is thus of great importance. Selection of insecticide-resistance variants has been shown to result in severe reduction of genetic diversity at loci involved during multiple insecticide-based interventions in both public health and agricultural sectors that have been conducted across Africa to control malaria vectors and crop pest insects [18,38]. *CYP6P9a* plays a key role in insecticide metabolic resistance in southern Africa in *An. funestus* [16,17,19] but whether the *CYP6P9a* resistant variant gene and others P450 insecticide resistance mechanisms remain confined to the southern part of Africa is an open question. In the present study, we sequenced a fragment of *CYP6P9a* gene and different loci spanning this gene and others resistance genes (*CYP6P9b, CYP6P4a, CYP6P4b)*, in a panel of different *An. funestus* populations across Africa. The goal was to establish the Africa-wide population structure of this major malaria vector, providing key evidence of the contemporary response of mosquito populations to insecticide interventions. 

### 4.2. Spread of Resistance in An. Funestus Populations Across Africa and Barriers to Gene Flow

We observed the predominance of one major haplotype made up of mosquito populations from Mozambique, Malawi, Zambia and Tanzania; southern African countries plus one Eastern African country. Taken together, our results support the possible presence of barriers to gene flow among mosquito populations from these four countries on one side, and central (DR Congo, Cameroon) and eastern (Uganda) African mosquito populations on the other. Based on phylogenetic relationships, haplotype diversities, *F_ST_* estimates and all other genetic differentiation indexes reported, southern African plus Tanzania populations are genetically differentiated from other populations at the *rp1* locus; among them, the resistance has spread and is now almost fixed. They always form a unique cluster distinct from those formed by central and eastern African mosquito populations. *An. funestus* mosquito populations from Mozambique and Malawi have previously been reported to share similar genetic differentiation patterns [16,18,19,39,40,41]. Our data show the resistance allele is fixed in southern Africa, and further highlights its expansion to Tanzania in eastern Africa where gene flow was not observed between Uganda and Tanzania. The fixation of the resistance in southern Africa is shown by the higher nucleotide diversity variability in *CYP6P9a* in Zambia in 2010 [40] at 0.04 versus 0.00042 reported here, suggesting a reduced role in resistance in the country at that time for this gene. We hypothesise that the presence of different geographical landforms like the African Great Lakes, mountain ranges and the Rift Valley in the region, are responsible for the lack of gene flow between southern Africa and other parts of the continent since the latter have been already been shown to affect genetic population structure in the major malaria vectors *An. funestus* and *An. gambiae* [42,43,44]. These patterns of genetic structure support the contrast in resistance patterns of *An. funestus* population across Africa [11,13,16,18,24,40,45,46] and also give an indication on the speed and the spread of the resistance on the continent. Indeed, the Rift Valley, if really a factor, may only slow the spread of the *CYP6P9a* resistance variant and not prevent it completely. Further monitoring is thus necessary especially since genome-wide microsatellite-based Bayesian clustering analysis data recently revealed Ugandan *An. funestus* mosquito population clustering with those from southern Africa, notably Mozambique and Zambia [47].

### 4.3. Pyrethroid Resistance in An. Funestus is Under Directional Selection

We confirmed a major selective sweep in the *rp1* region which is one of the major pyrethroid resistance QTL where P450 genes, including *CYP6P9a*, associated with metabolic pyrethroid resistance have been identified [17,19]. Overall, multiple analyses provide evidence for a selective sweep occurring in this QTL were supported by a reduction in the genetic variation in mosquito populations from southern and part of eastern Africa (Figure 2, Figure 3 and Figure 4; Table 1), of *CYP6P9a* key insecticide resistance genes as well as others fragments from the BAC clone in the vicinity of the gene. It is important to mention that these BAC fragments, in addition to overlap *CYP6P9a* gene locus, also covers genes like *CYP6P9b*, *CYP6P4a*, *CYP6P4b*, located in the same *rp1* region and that have already been identified to play an important role in pyrethroid resistance in *An. funestus* [17].

#### 4.3.1. CYP6P9a Resistance Allele Has Moved Beyond Southern Africa into Eastern Africa

Strong directional selection acting on *CYP6P9a* was observed in Mozambique, Malawi, Zambia and Tanzania, mirroring the predominant role of cytochrome P450 genes in the metabolic resistance observed in *An. funestus* population as previously reported in southern Africa [16,40] and in other mosquito species such as *An. gambiae* [48] and *Aedes* mosquitoes [29,49]. We found that almost all SNPs in coding sequence of the first exon of *CYP6P9a* that occurred in Mozambique, Malawi, Zambia and Tanzania were shared and non-synonymous (Figure S2), suggesting that *CYP6P9a* is under positive selection in these four countries. The reduced haplotype diversity of *CYP6P9a* gene with almost no mutational steps between the four countries is consistent with positive selection occurring. The selection of this *CYP6P9a* has been shown to be metabolically more efficient in metabolising pyrethroids [50], which may explain why pyrethroid resistance has been continuously associated with a high overexpression of *CYP6P9a* and *CYP6P9b* genes in multiple *An. funestus* populations from southern Africa [16,19,39,40,45]. However, the expression levels of key P450s such as *CYP6P9a* and *CYP6P9b* varies between populations suggesting that independent selection events for resistance to pyrethroids has occurred [41]. In this study, Cameroon, DRC and Uganda displayed higher diversity at this locus, showing that this P450 resistance mechanism may play a lesser role in conferring pyrethroid resistance to *An. funestus* population from these regions where *CYP6P9a* has also been reported to be overexpressed but to at a lower level than southern Africa [24,29,40,41] as further supported by their higher genetic diversity in Uganda than in Malawi but also than in West (Benin and Ghana) and Central Africa (Cameroon) [18].

Distinct genes associated with insecticide resistance, including the glutathione S-transferase *GSTe2* and the Cytochrome P450 *CYP9K1* genes, have been found to be more overexpressed in West/Central, East Africa respectively [19,24,41,51]. Positive selection acting on *CYP6P9a* is similar to that of *GSTe2* conferring DDT (DichloroDiphenylTrichloroethane) resistance in *An. funestus* populations West/Central Africa. A clear example is that provided by the resistant samples from Benin exhibiting a *GSTe2*-derived resistant haplotype which is almost fixed while DDT susceptible populations remained highly polymorphic at this locus in 2014 [51].

Patterns of genetic differentiation based on *CYP6P9a* showed southern African plus Tanzanian mosquito populations to be significantly differentiated from populations from other regions. Again, this pattern mimics the one of *GSTe2* gene in west Africa and further supports the presence of barriers to gene flow between southern Africa plus Tanzania on one side and Uganda plus Central Africa on the other side. It is important to note that Tororo, the collection site in Uganda, is surrounded by east and west branches of the African rift Valley and is located north of Lake Victoria on the border with Kenya. The predominant *CYP6P9a* haplotype formed by southern African plus the Tanzanian mosquito population was completely absent from the other countries in line with the low level of gene flow observed between the two defined regions. This haplotype is a resistant allele similar to a *CYP6P9a* resistant cluster from Mozambique and Malawi [16,18,40] that were shown to have an increased efficiency to metabolise pyrethroids [40]. Indeed, insecticide-based interventions relying mainly on LLINs and IRS implemented in these countries clearly impacted vector adaptation behaviour leading to resistance selection [7,18,52,53].

In contrast to previous reports [13,18,40,41], the *CYP6P9a* resistance allele has moved from southern Africa to part of eastern Africa but has not (yet) crossed the rift valley into Uganda. This contrasting distribution mirrors the distribution of the *GSTe2* resistance allele which is completely absent from southern Africa and present in Uganda [51]. There is a need for a continuous monitoring of the resistance spread across Africa to ensure the role of the rift valley playing a role in the observed restriction to gene flow. Examples of resistance alleles that were initially confined within a certain area before spreading beyond include the *296S* resistant allele initially located only in West to East Africa and completely absent from southern Africa in 2010 [46] that has now been detected in southern Africa [15]. The same is true for knockdown resistance (*kdr*) mutations in other mosquito species such as *An. gambiae* [54] or *Ae. aegypti* [55].

#### 4.3.2. Reduced Polymorphism of the 120 kb rp1 Genomic Region Show a Selective Sweep in the Vicinity of *CYP6P9a*

Polymorphism analysis of the *rp1* QTL in *An. funestus* population across Africa found reduced polymorphisms around *CYP6P9a* in almost all populations except in those from central Africa. In general, the selection on the *rp1* region appears to be more extensive in southern and parts of eastern Africa in line with previous reports [16,18,53]. The reduced diversity that was observed in Mozambique, Malawi, Zambia and Tanzania as well as the reduced haplotype diversity are due to a selective sweep that was driven by effort to control the mosquito population as recently shown that a single cytochrome P450 resistance allele (CYP6P9a_R) in *An. funestus* reduced the efficacy of insecticide-treated bed nets in southern Africa [19]. In these four countries, selection is more pronounced in the vicinity of *CYP6P9a* from −9 kb upstream the gene to +29 kb downstream, where lower levels of polymorphism were recorded (Figure 3b). At loci +61 kb and +86 kb farther, low polymorphism was also observed (Figure 3b). In the remaining locus, −34 kb (Figure 3a), the diversity was still lower compared to other parts of Africa (but not reflected by the relative diversity results) showing that the region under selection could be greater than the 120 kb region studied. This profile is attributable to the proximity of the un-polymorphic loci to key P450 resistance genes and can be explained by the fact that in a situation of near fixation of a selective sweep as seen in this study for southern and Tanzanian populations, directional selection is indicated by reduced genetic variation [56]. In eastern Africa, a similar selective sweep profile was observed around −9 kb and +61 kb reflecting the different resistance profiles we identified. In central Africa, except at the level of locus +86 kb where low polymorphism may indicate selection of some kind, no selective sweep was observed. A locus encoding HEXIM 1/2 protein (*AFUN008365*), overlaps +86 kb locus (Table S6). This protein’s function is not yet characterised in *An. funestus* but has been reported to act as a transcriptional regulator which functions as a general RNA polymerase II transcription inhibitor in humans [57,58]. One can thus hypothesise that *AFUN008365* gene may play a certain role in resistance development in +86 kb locus. This result is in line with the resistance patterns we described in the region. Based on haplotype networks, we showed a Kinshasa, DRC *An. funestus* population that was clustering with the major resistant haplotype from southern Africa at locus +86 kb (120 BAC), suggesting the arrival of a southern resistance allele in central Africa. The resistance allele in question was not identified in Kinshasa, hence the possible existence of a further barrier that crosses DRC. The polymorphism pattern at locus +86 kb in DRC could also be evocative of the presence of a different resistance patterns occurring at this level as supported by the recent characterisation of another resistance marker located in the 3L chromosomal region spanning the *rp3* QTL of *An. funestus* genome; the P450 pyrethroid metabolizer *CYP9J11* gene which is commonly overexpressed in all countries across Africa [41]. Since in southern Africa the region under selection spans from −9 kb from *CYP6P9a* and beyond +86 kb, implying it is greater than the 120 kb region studied, it is necessary to analyse chromosomal-level regions using the recently available *An. funestus* genome assembly [59] to clearly demarcate the extent of this selective sweep. 

## 5. Conclusions

This study provides evidence for the spread of a selective sweep on a cytochrome P450 responsible for metabolic resistance to insecticides in the major malaria vector *An. funestus*. Through several analytical approaches, our results show that the P450-based resistance mechanism is not confined to the southern part of Africa but is now fixed there and has spread to Tanzania in eastern Africa. Our findings are also supportive of the presence of barriers to gene flow between populations of *An. funestus* which are likely to prevent or slow the spread of the CYP6P9-based resistance mechanism from southern Africa to other parts of Africa. We identified that the rift valley region probably acts as a strong barrier to gene-flow, stressing the importance of continuous monitoring of major vectors population structure for vector control programmes. Finally, we highlight that, despite the heterogenous population structure of *An. funestus* across Africa being favourable to resistance management, it will be a challenge to the deployment of any genetic-based vector control approaches such as gene-drive mechanisms. We provide vital information that should be taken into account in resistance monitoring as well as in the implementation of novel vector control strategies that rely on populations intermixing freely.

## Figures and Tables

**Table 1:** Nucleotide diversity and neutral test at CYP6P9a.

	2n	S	h	hd	π	D	D *	FS
Complete sequence fragment (1139 bp)								
Mozambique_Palmeira	20	1	2	0.10	0.00009	−1.16	−1.54	−0.88
Mozambique_Magania	32	3	3	0.12	0.00016	−1.73	−2.73 *	−1.71
Mozambique	52	4	4	0.11	0.00014	−1.86 *	−3.43 **	−3.63 *
Malawi	28	7	8	0.59	0.00061	−1.84 *	−1.46	−5.83 **
Zambia	20	3	3	0.28	0.00042	−1.16	−0.12	−0.29
Tanzania_Muheza	16	3	3	0.24	0.00033	−1.70	−2.20	−0.90
Tanzania_Tulizamoyo	24	3	3	0.24	0.00035	−1.26	−0.19	−0.50
Tanzania_Ikwambi	18	1	2	0.11	0.00010	−1.16	−1.50	−0.79
Tanzania	58	7	6	0.20	0.00027	−2.05 *	−2.77 *	−4.89 *
Uganda	18	9	6	0.56	0.00105	−1.92 *	−2.11	−1.91
DR Congo	26	24	9	0.53	0.00200	−2.33 **	−2.90 **	−2.04
Cameroon	20	23	7	0.68	0.00304	−1.79	−1.93	0.44
Overall	222	84	39	0.61	0.01095	−0.42	−3.96 **	−0.53
Coding Region (642 bp)								
Mozambique_Palmeira	20	1	2	0.10	0.00016	−1.16	−1.54	−0.88
Mozambique_Magania	32	3	3	0.12	0.00029	−1.73	−2.73 *	−1.71
Mozambique	52	4	4	0.11	0.00024	−1.86 *	−3.43 *	−3.63 *
Malawi	28	2	3	0.27	0.00043	−0.97	0.82	−1.09
Zambia	20	0	1	0.00	0.00000	-	-	-
Tanzania_Muheza	16	3	3	0.24	0.00058	−1.70	−2.20	−0.90
Tanzania_Tulizamoyo	24	0	1	0.00	0.00000	-	-	-
Tanzania_Ikwambi	18	1	2	0.11	0.00017	−1.16	−1.50	−0.79
Tanzania	58	4	4	0.10	0.00021	−1.85 *	−3.51 **	-3.84 *
Uganda	18	0	1	0.00	0.00000	-	-	-
DR Congo	26	6	4	0.22	0.00083	−1.96 *	−2.52 *	−1.19
Cameroon	20	16	6	0.62	0.00403	−1.58	−1.37	0.47
Overall	222	41	20	0.54	0.01328	0.54	−2.42 *	4.08 *
Non-Coding Region (497 bp)								
Mozambique_Palmeira	20	0	1	0.00	0.00000	-	-	-
Mozambique_Magania	32	0	1	0.00	0.00000	-	-	-
Mozambique	52	0	1	0.00	0.00000	-	-	-
Malawi	28	5	6	0.39	0.00085	−1.86 *	−2.30	−4.66 **
Zambia	20	3	3	0.28	0.00096	−1.16	−0.12	−0.29
Tanzania_Muheza	16	0	1	0.00	0.00000	-	-	-
Tanzania_Tulizamoyo	24	3	3	0.24	0.00081	−1.26	−0.19	-0.50
Tanzania_Ikwambi	18	0	1	0.00	0.00000	-	-	-
Tanzania	58	3	3	0.10	0.00034	−1.47	−0.45	−1.60
Uganda	18	9	6	0.56	0.00242	−1.92 *	−2.11	−2.11
DR Congo	26	18	8	0.47	0.00353	−2.24 **	−2.61 *	−2.07
Cameroon	20	7	6	0.45	0.00175	−1.84 *	−2.46 *	−2.70 *
Overall	222	43	28	0.54	0.00790	−1.34	−4.28 **	−6.63 **

Column headings: 2n: number of sequences; S: segregating sites; h: number of haplotypes; hd: haplotype diversity; π: Nucleotide diversity (Pi); D: Tajima’s D; D*: Fu and Li’s D* statistic; FS: Fu’s FS test. (*) represent significant results and level of significance: * = *p* ≤ 0.05; ** = *p* ≤ 0.01.

**Table 2:** Polymorphism rate measured by pN/pS ratios of the CYP6P9a locus for seven countries.

	Synonymous Sites	Synonymous Substitution	Synonymous Polymorphism Per Site (pS)	Non-Synonymous Sites	Non-Synonymous Substitution	Non-Synonymous Polymorphism Per Site (pN)	Polymorphism Rate (pN/pS)
Mozambique	153.47	1	0.007	488.53	3	0.006	0.94
Malawi	153.43	0	0.000	488.57	2	0.004	-
Tanzania	153.50	0	0.000	488.50	0	0.000	-
Zambia	153.53	2	0.013	488.47	2	0.004	0.31
Uganda	155.17	0	0.000	486.83	0	0.000	-
DR Congo	157.09	1	0.006	484.91	5	0.010	1.62
Cameroon	156.63	0	0.000	485.38	16	0.033	-

pN/pS < 1: Purifying selection; pN/pS > 1: positive selection; pN/pS = 1: Neutrality.

**Table 3:** Nucleotide diversity and neutral test at CYP6P9a and at 5 loci around it across Africa.

	Position (kb)	Length (bp)	Mozambique			Malawi			Zambia			Tanzania			Uganda			DR Congo			Cameroon			Pi Ratio *
			hd	π*10E3	D	hd	π*10E3	D	hd	π*10E3	D	hd	π*10E3	D	hd	π*10E3	D	hd	π*10E3	D	hd	π*10E3	D	
0BAC	−34	626	0.04	0.06	−1.10	0.31	0.72	−1.43	0.22	0.36	−1.22	0.36	1.08	−2.18 **	0.36	1.76	−1.61	0.15	0.74	−2.09 *	0.91	6.49	−0.80	4.84
25BAC	−9	754	0.04	0.14	−1.68	0.38	1.27	−1.24	0.21	0.94	−2.20 **	0.23	1.28	−1.92 *	n.a	0.00	n.a	0.13	0.35	−1.89 *	0.86	5.53	−0.54	1.62
CYP6P9a	0	1139	0.11	0.14	−1.86 *	0.59	0.61	−1.84 *	0.28	0.42	−1.16	0.20	0.27	−2.05 *	0.56	1.05	−1.92 *	0.53	2.00	−2.33 **	0.68	3.04	−1.79	1.41
63BAC	+29	614	0.43	1.11	−0.68	-	-	-	-	-	-	0.64	1.81	1.05	0.60	1.10	0.18	0.46	1.99	-1.68 *	-	-	-	1.06
95BAC	+61	671	0.41	1.24	−1.38	0.20	0.30	−1.51	0.79	1.76	−1.03	0.26	0.74	−1.85 *	0.33	1.19	−1.93 *	0.24	0.62	−1.70	0.85	4.27	−1.66	1.80
120BAC	+86	633	0.11	0.17	−0.81	n.a	0.00	n.a	0.07	0.19	−1.49	0.37	4.94	−2.09 *	0.39	2.06	−1.83 *	n.a	0.00	n.a	0.46	3.12	−1.76 *	1.37

Column headings: hd: haplotype diversity; π: Nucleotide diversity (Pi); D: Tajima’s D; Pi ratio: ratio between east/central and southern Africa. (*) represent significant results and level of significance: * = *p* ≤ 0.05; ** = *p* ≤ 0.01.

## Supplementary Materials

The following are available online at https://www.mdpi.com/2073-4425/11/11/1314/s1, Figure S1: Amplification and sequencing scheme of tthe key insecticide resistance gene CYP6P9a (a) and the BAC clone (b) Figure S2: Schematic position of nucleotide polymorphisms at *CYP6P9a* and haplotypes across the gene. Figure S3: Phylogenetic trees generated by the Maximum likelihood method based on the Hasegawa-Kishino-Yano model. Figure S4: Haplotype network for nucleotide Non-synonymous variants in 6p9a resistance gene. Figure S5: Levels of nucleotide diversity survey at *CYP6P9a* and five loci around the pyrethroid resistance gene. Figure S6: Ratio of nucleotide diversity at *CYP6P9a* and five loci around the pyrethroid resistance gene. Figure S7: Phylogenetic trees generated with Maximum Likelihood method on *A. funestus* BAC clone from 7 sub-Saharan African countries. Figure S8: Haplotype network for nucleotide variants in BAC fragments in 7 sub-Saharan African countries. Figure S9: Phylogenetic tree generated by the Neighbor-Joining method based on pairwise *Fst* distance. Table S1: Country and sampling sites. Table S2: List of BAC clone and *CYP6P9a* primers used for PCRs and sequencing. Table S3: Gene flow and genetic differentiation patterns. Table S4: Pairwise Fst distance based on 6p9a sequences from different countries. Table S5: Ratio of nucleotide diversity at *CYP6P9a* and five loci around the pyrethroid resistance gene. Table S6: Location of different BAC fragments regarding *An. funestus* genome annotation features from vector base Table S7: Gene flow and genetic differentiation patterns Table S8: Pairwise Fst distance between different countries.
