# Supplementary material for: CYP6P9-Driven Signatures of Selective Sweep of Metabolic Resistance to Pyrethroids in the Malaria Vector Anopheles funestus Reveal Contemporary Barriers to Gene Flow
Source: Genes (Basel). 2020 Nov 5;11(11):1314. doi: 10.3390/genes11111314 (PMC7694540; doi:10.3390/genes11111314)
Supplement: Supplementary file 1 [file genes-11-01314-s001.pdf]

Supplemental Data

Figures

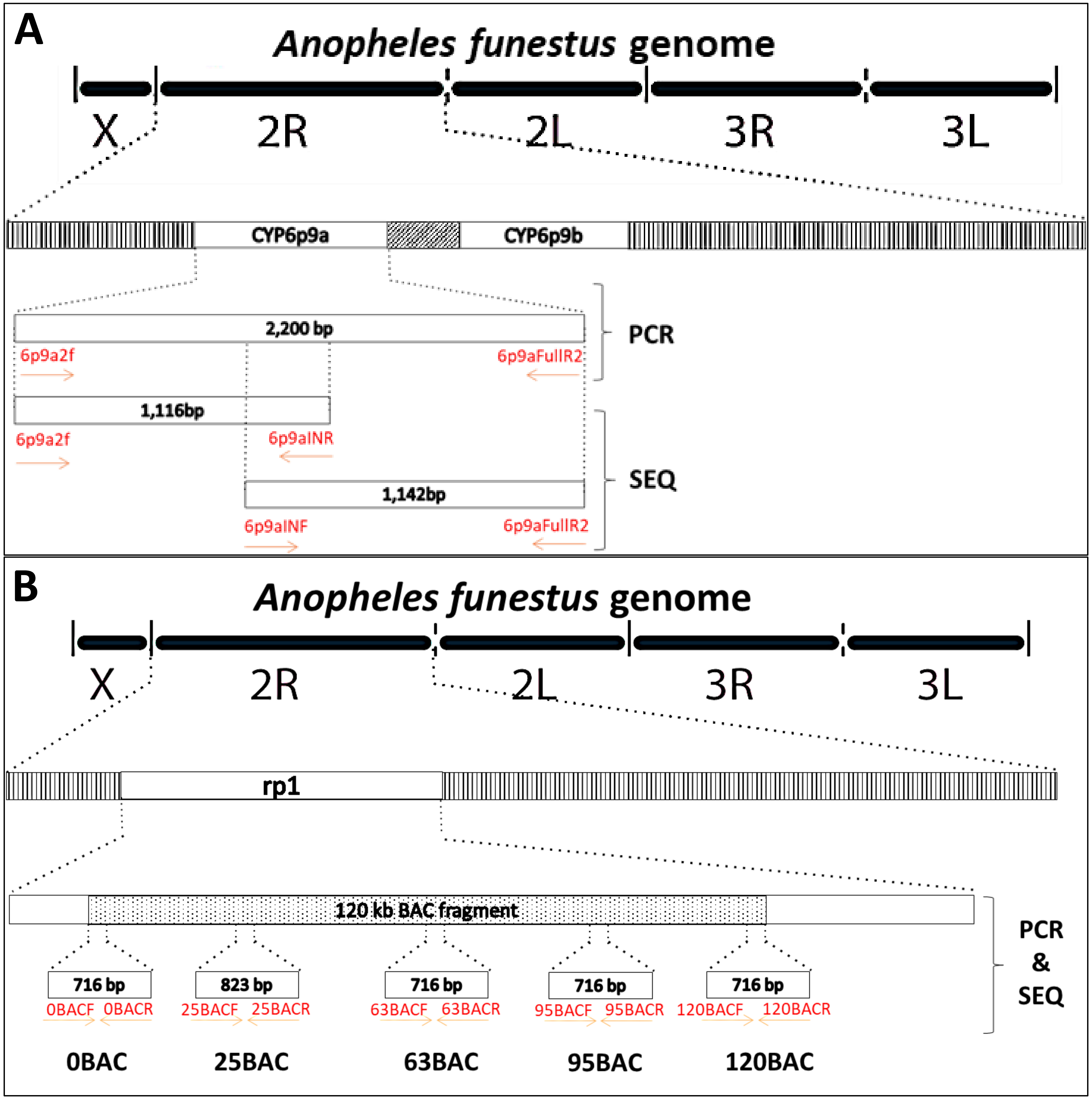

Fig. S1: amplification and sequencing scheme of tthe key insecticide resistance gene CYP6P9a (A) and the BAC clone (B)

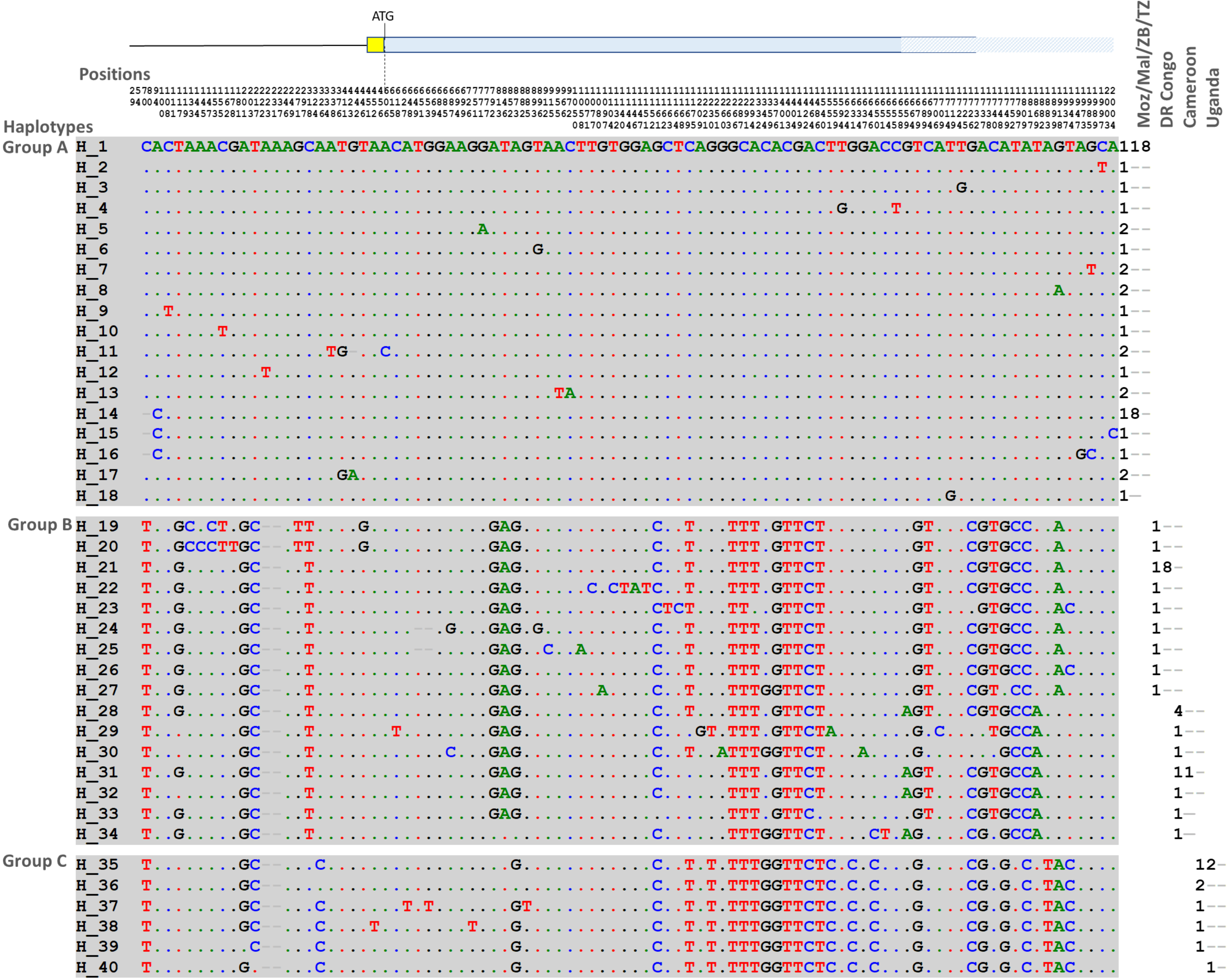

Fig. S2: Schematic position of nucleotide polymorphisms at *CYP6P9a* and haplotypes across the gene.

Nucleotide polymorphism was examined across the 1,139 bp of *CYP6P9a* gene including successively, 465 bp of the partial 5' upstream intergenic region (black line), 32 bp of 5'UTR (yellow box) and 642 bp of partial gene's first exon (blue box),. The number below the model indicate each nucleotide position on the sequence. In each haplotype, letters indicate alternative nucleotide at given position with reference to haplotype 1. The short line dash in haplotypes represents deletions. Numbers on the left-hand side of the figure indicate the number of sequences contained in each haplotype.

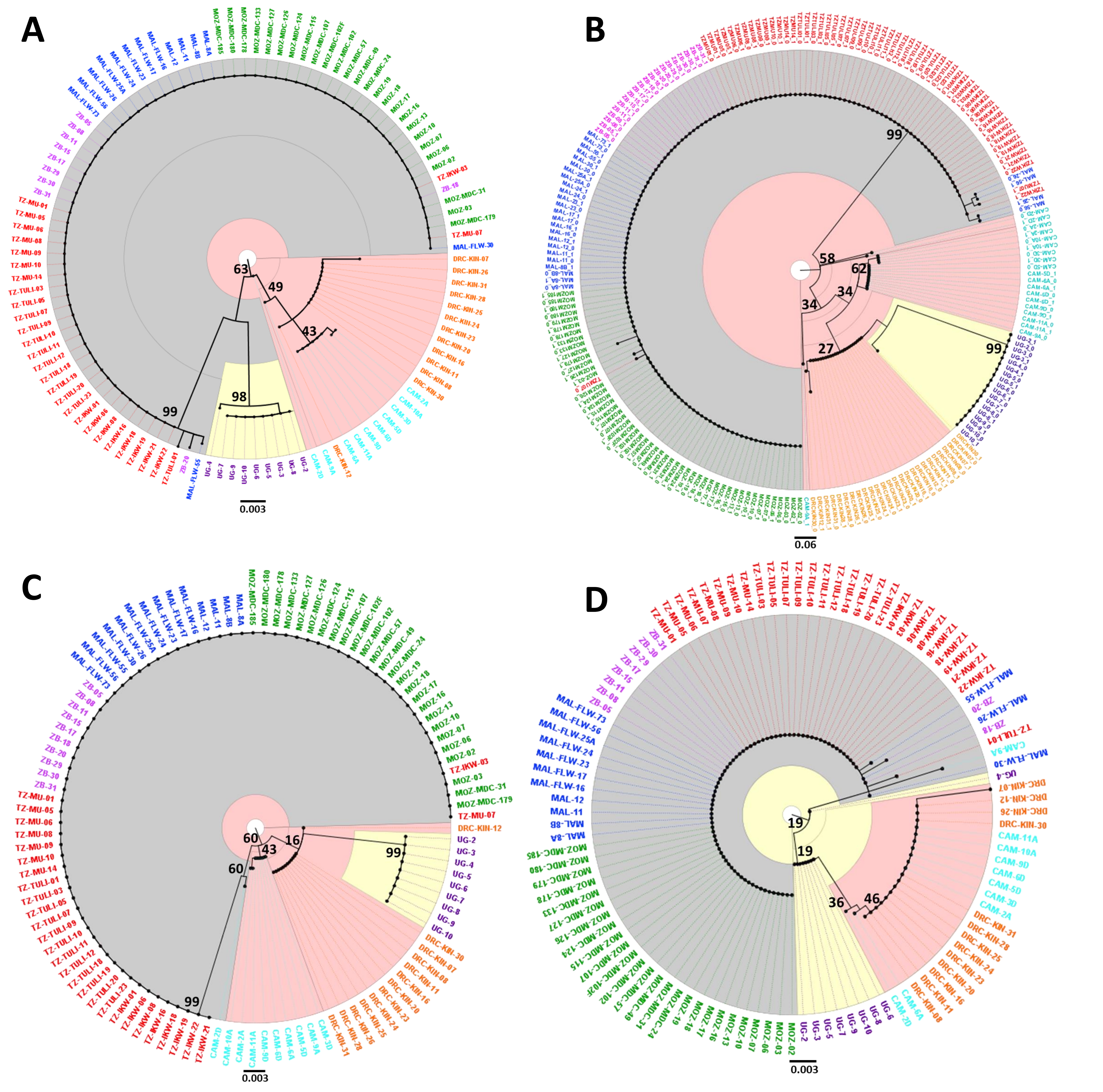

Fig. S3 : Phylogenetic trees generated by the Maximum likelyhood method based on the Hasegawa-Kishino-Yano model inferred on a 1,139-bp fragment of CYP6P9a gene (A), the 36 non-synonymous mutations found in the fragment (B), 498-bp fragment of CYP6P9a gene's coding region (C) and 461-bp of the gene's non-coding region (D). All but one trees include 111 *A. funestus* sequences from 7 sub-Saharan African countries generated in this work including 10 reference sequences from Cameroon and 9 from Uganda. Non synonymous mutations tree include 222 *A. funestus* unphased sequences. Trees with the highest log likelihood are shown. To test the robustness of each tree topology, 1,000 bootstrap replicates were performed. Trees are drawn to scale, with branch lengths measured in the number of substitutions per site.

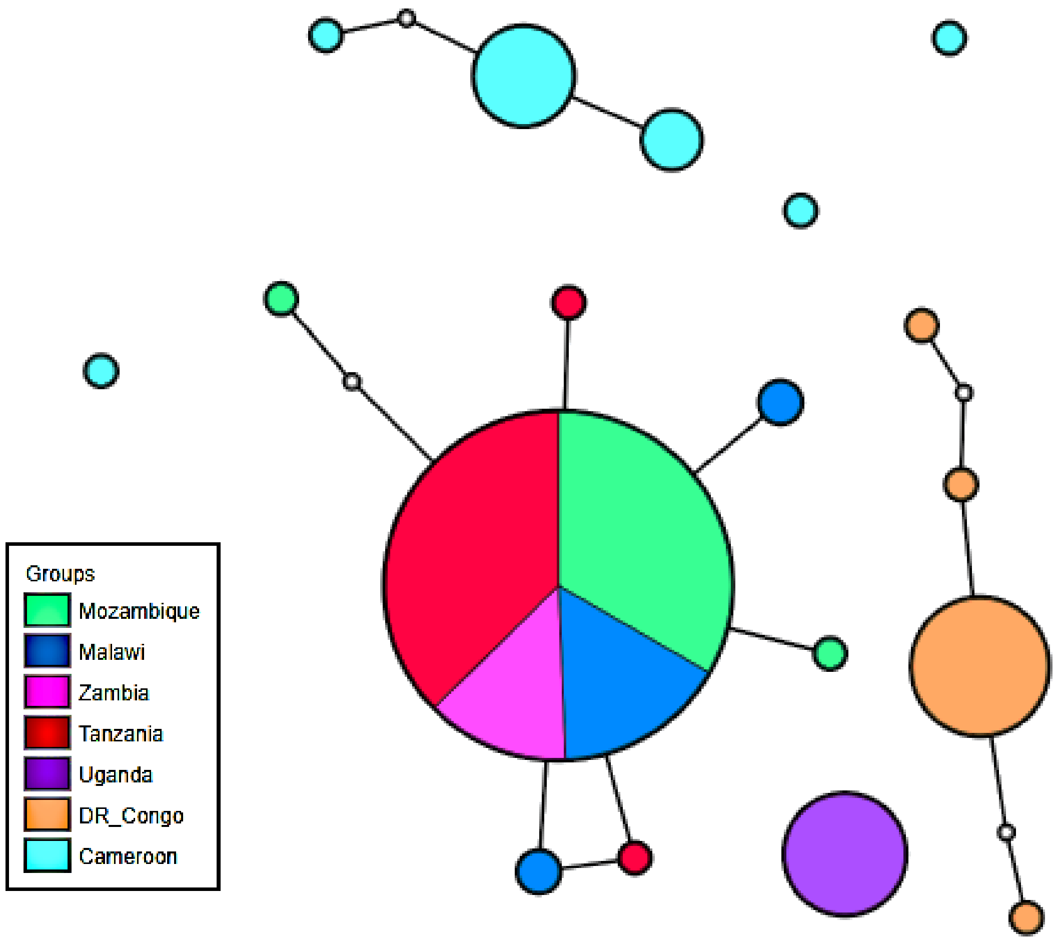

Fig. S4 : Haplotype network for nucleotide Non-synonymous variants in *6p9a* resistance gene. The green, Bleu, red and Pink parts of the circle constitute the major haplotype respectively shared between Mozambique (MOZ), Malawi (MAL), Zambia (ZB) and Tanzania (TZ). The Orange, Cyan and Purple circles shows three distinct haplotypes representing mosquitoes population from Democratic Republic of Congo (DRC), Cameroon (CAM) and Uganda (UG) respectively. The size of the shapes is proportional to the frequency of the haplotype and spots on each branch show the mutational steps separating haplotypes.

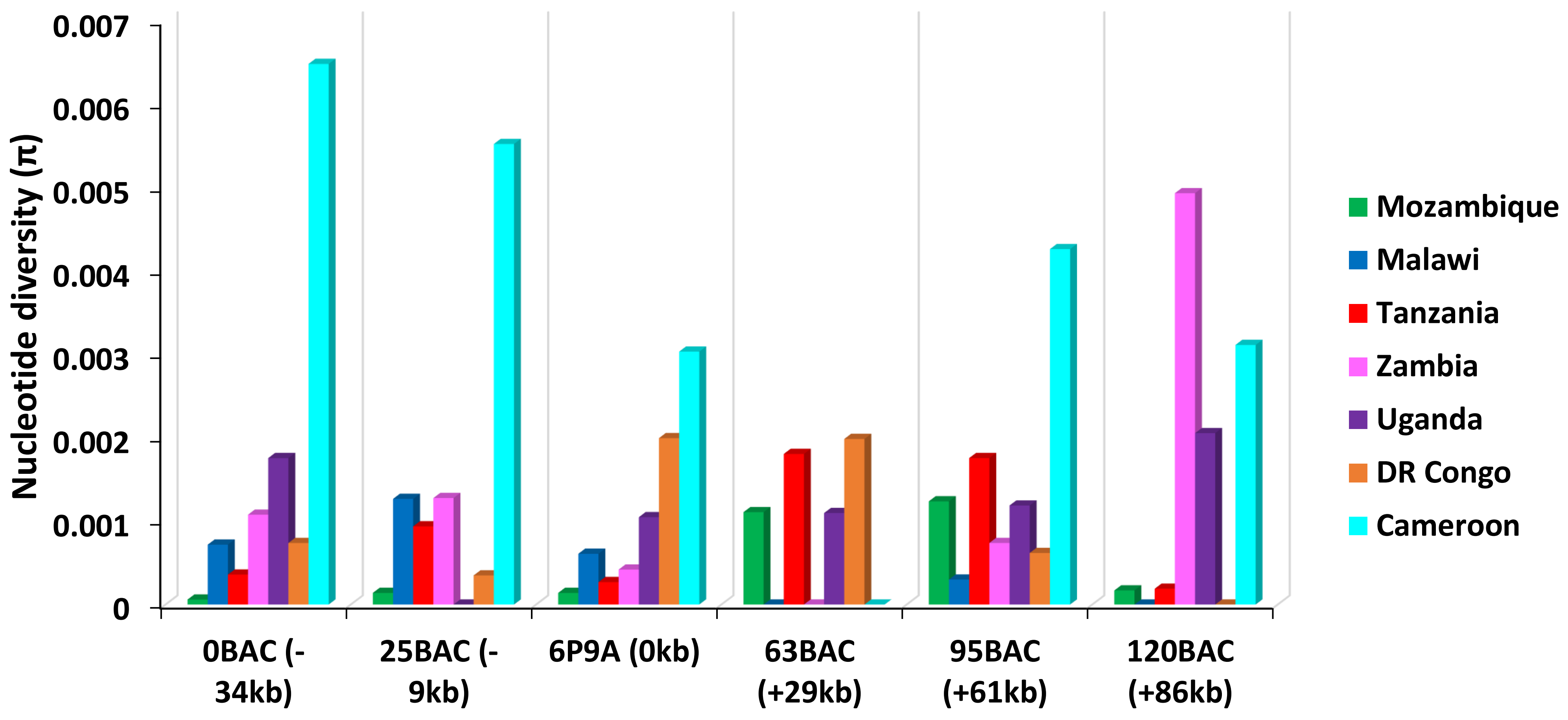

Fig. S5: Levels of nucleotide diversity survey at *CYP6P9a* and five loci around the pyrethroid resistance gene. For each locus, nucleotide diversity was investigated by sequencing *A. funestus* samples from seven sub-Saharan African countries. Positions (kb) of sequenced regions relative to CYP6P9a.

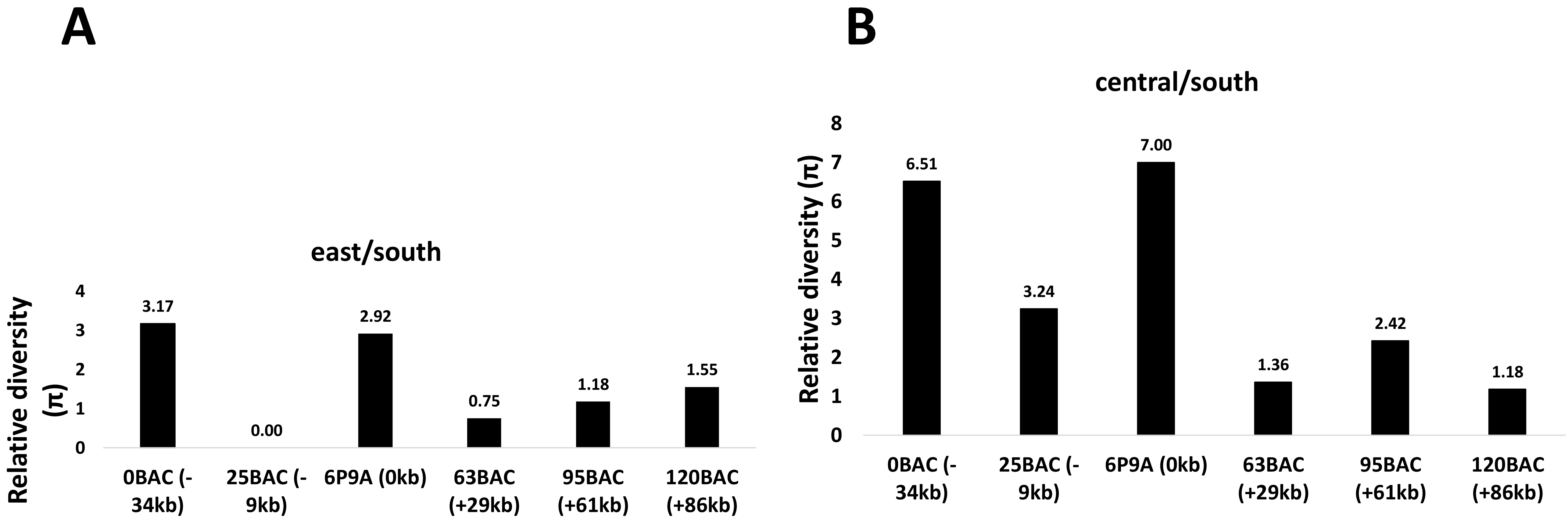

Fig. S6: Ratio of nucleotide diversity at *CYP6P9a* and five loci around the pyrethroid resistance gene. For each locus, nucleotide diversity was investigated by sequencing *A. funestus* samples from seven sub-Saharan African countries. Results were further grouped samples into Southern, Eastern and Central Africa respective to mosquitoes field population origin. Positions (kb) of sequenced regions relative to CYP6P9a. Ratio of relative nucleotide diversity at each loci was calculated between Eastern and Southern Africa **(A)**, and Central and Southern Africa **(B)**.

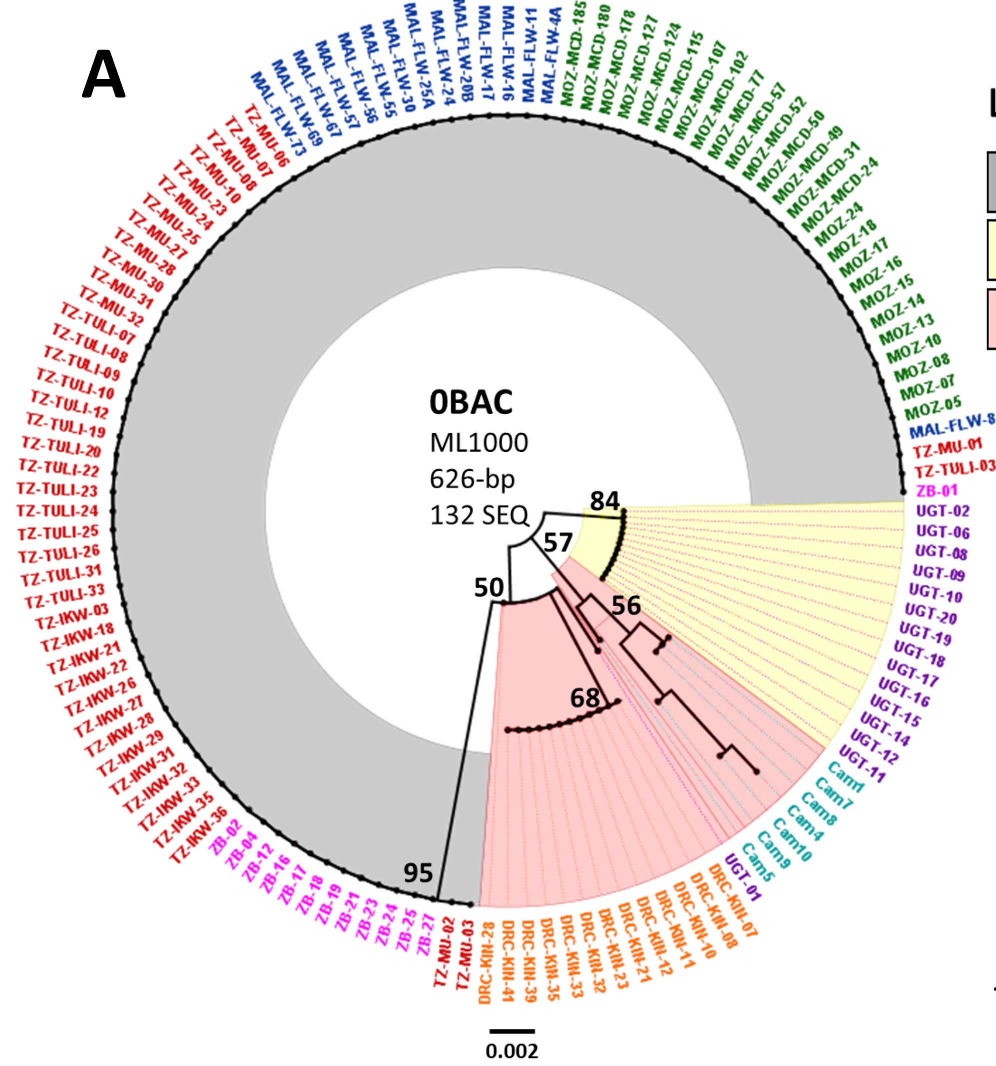

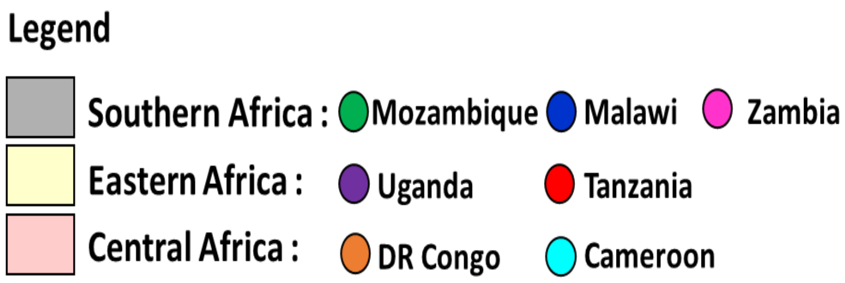

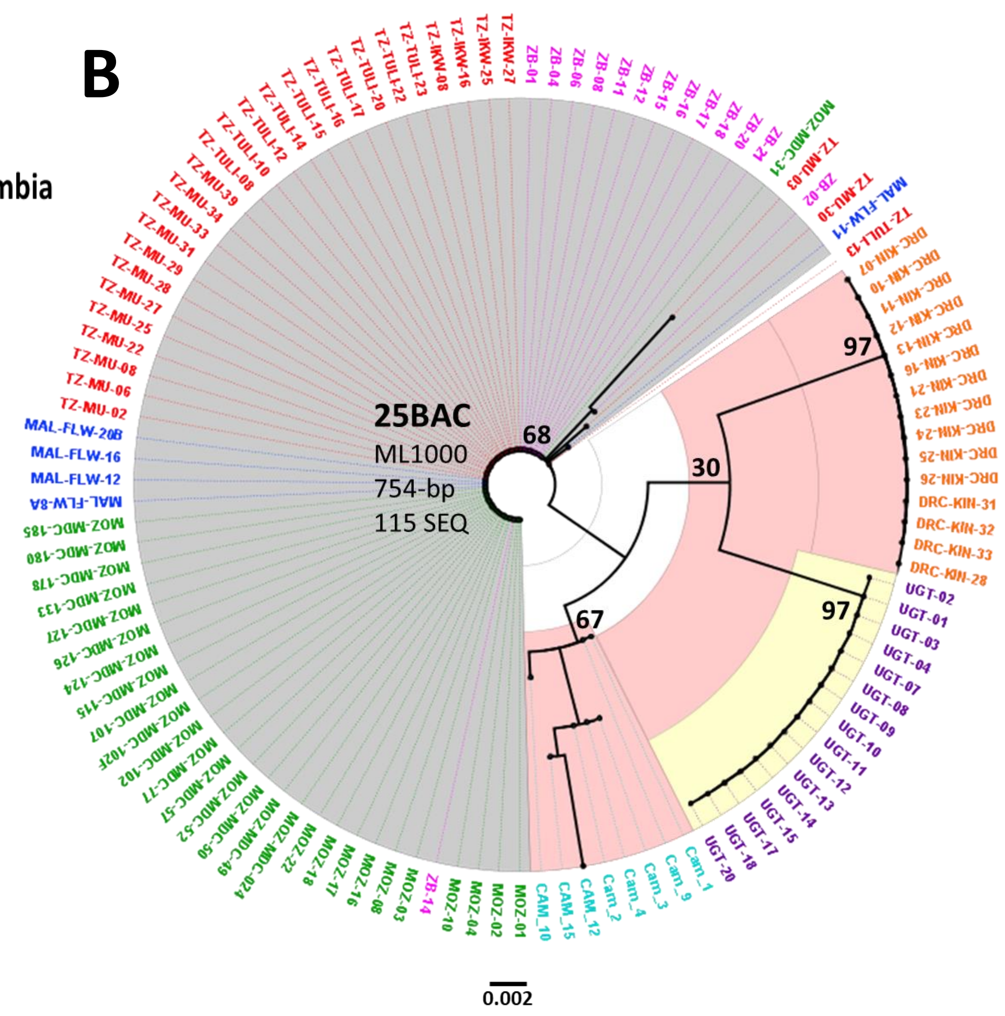

Fig. S7: Phylogenetic trees generated with Maximum Likelihood method on *A. funestus* BAC clone from 7 sub-Saharan African countries.

Final alignments were submitted to MEGAX ModelTest program to select the best model according to the Akaike Information Criterion. The Hasegawa-kishino Yano Model plus a gamma distribution was inferred on 132 *A. funestus* 0BAC fragment sequences measuring 626-bp in length **(A)**, 115 *A. funestus* 25BAC fragment sequences measuring 754 bp **(B)** and 47 *A. funestus* 63BAC fragment sequences measuring 614bp **(C)**. The Kimura-2 model was inferred on 121 95BAC fragment sequences measuring 671bp **(D)**. To test the robustness of each tree topology, 1000 bootstrap replicates were calculated and the numbers at some nodes of the tree indicate frequencies of occurrence for 100 trees. Trees with the highest log likelihood are shown. The branch lengths are drawn to scale, and the scale bar indicates nucleotide replacements per site**.**

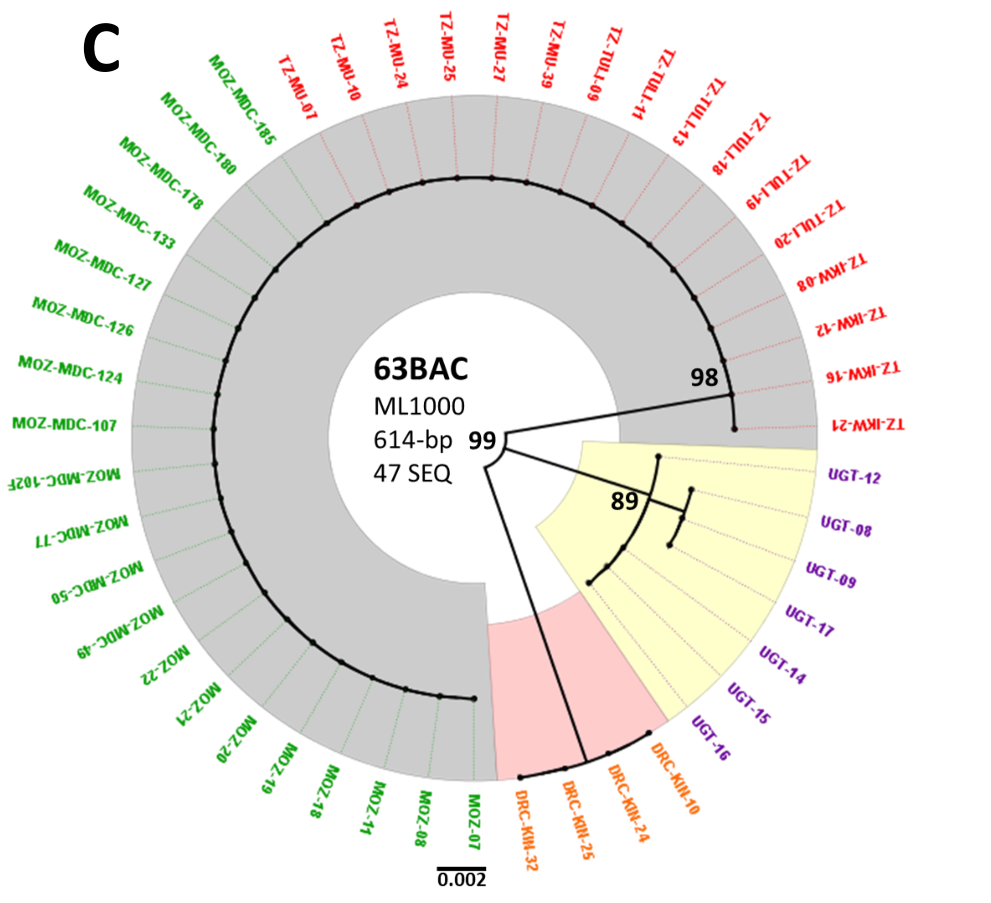

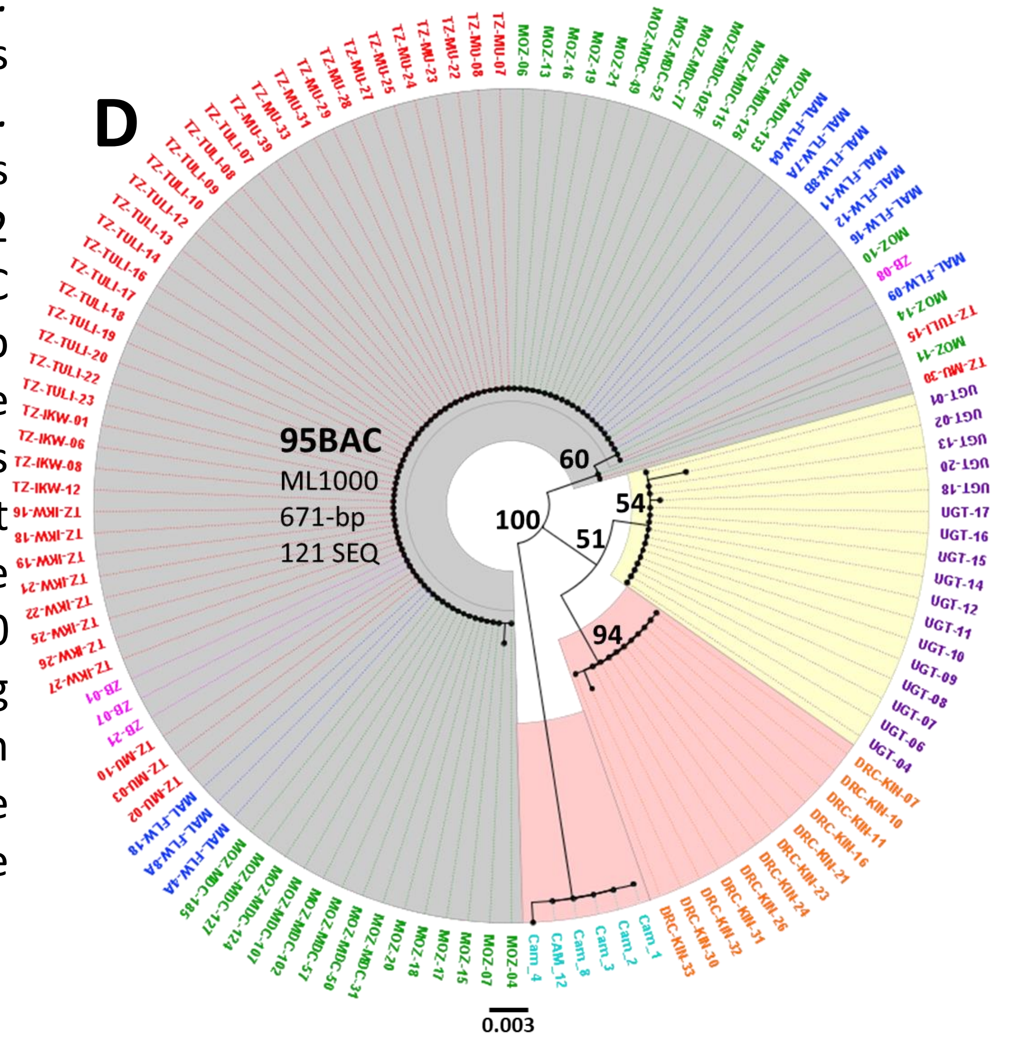

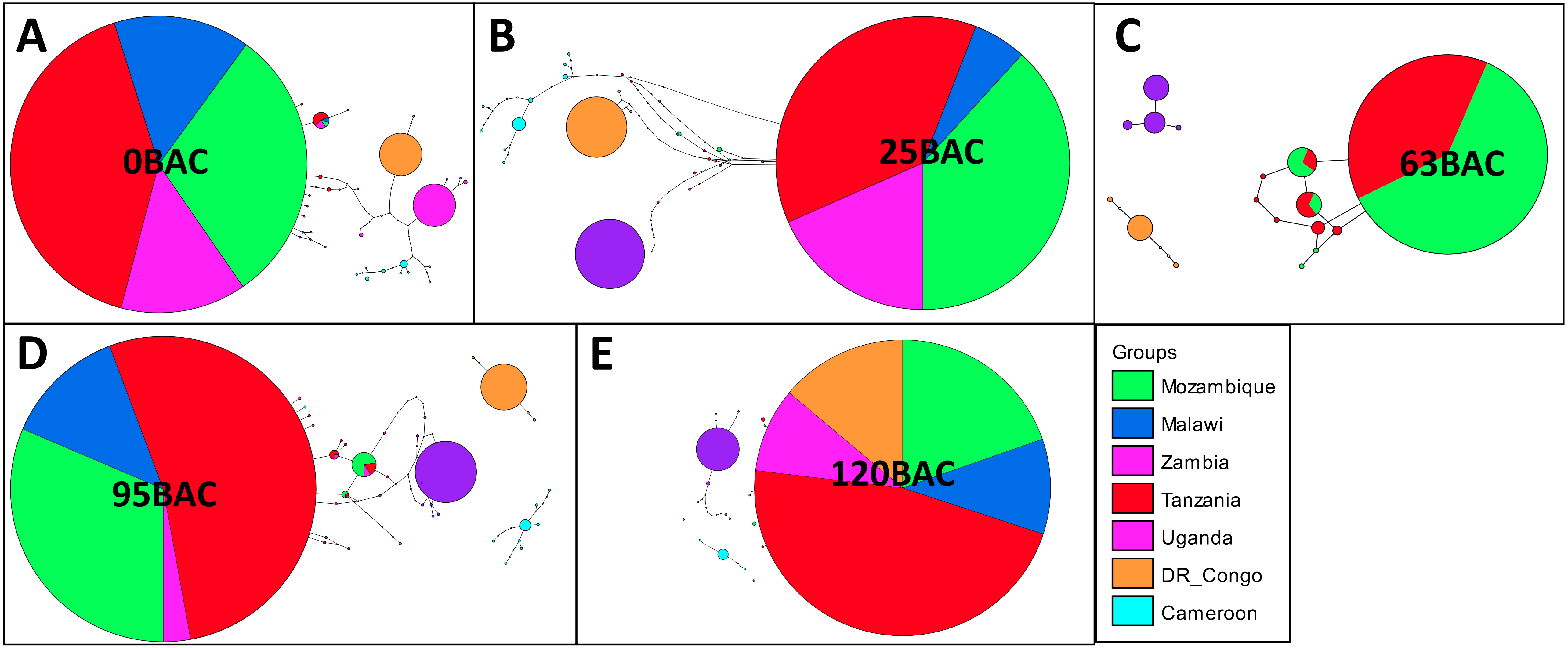

Fig. S8 : Haplotype network for nucleotide variants in BAC fragments in 7 sub-Saharan African countries

Africa-wide TCS network for the 0BAC **(A)**, 25BAC **(B)**, 63BAC**(C)**, 95BAC **(D)** and 120BAC **(E)** haplotypes showing predominant haplotypes in southern Africa and Tanzania later Joined by DR Congo. A nearly fixed haplotype is seen in Uganda (Purple) and DR Congo (Orange). Cameroon (Cyan) show a greater diversity. Lines connecting haplotypes and each node represent a single mutation event.

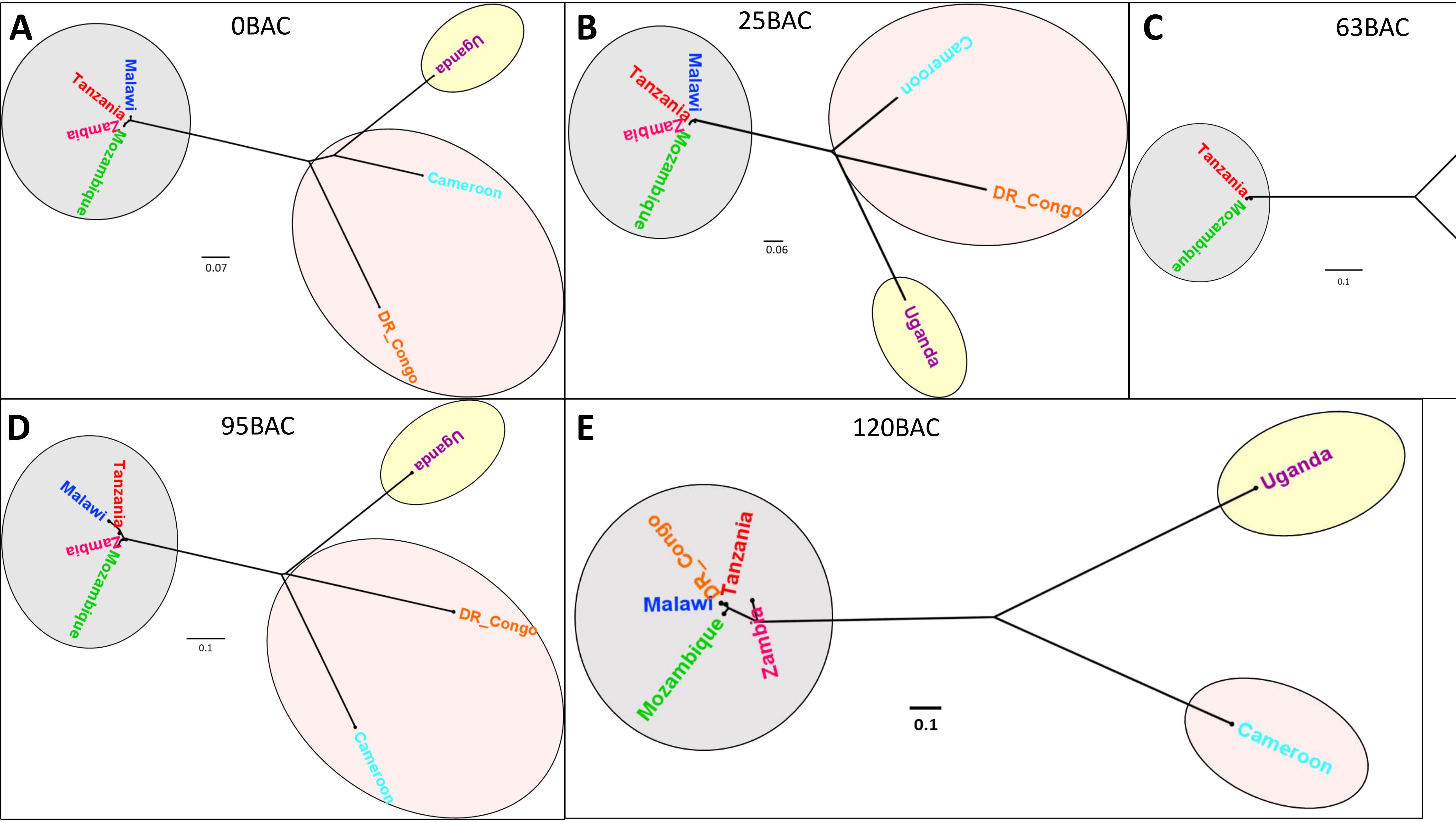

Fig. S9: Phylogenetic tree generated by the Neighbor-Joining method based on pairwise *Fst* distance

Trees based on F_{st} distance data were computed from *A. funestus* 0BAC fragment sequences **(A)**, 25BAC fragment sequences **(B)**, 63BAC fragment sequences **(C)**, 95BAC fragment sequences **(D)** and 120BAC fragment sequences **(E)** from seven African countries, grouped, respective to each samples origin, into Southern Africa (Mozambique, Malawi, Zambia), Eastern Africa (Tanzania and Uganda) and Central Africa (DR Congo and Cameroon). Optimal trees with the sum of branch length is shown. Trees are drawn to scale, with branch lengths in the same units as those of the evolutionary distances used to infer the phylogenetic tree.

Tables

Table S1: Country and sampling sites

Countries	Sites	Coordinates	Collection date
Mozambique	Palmeira	25° 15’ 19” S<br>32° 52’ 22” E	April 2017
	Magania da Costa	17° 18' 40" S<br>37° 30' 53" E	March 2013
Malawi	Fulirwa	9° 55' 59.99" S<br>33° 55' 59.99" E	February 2017
Zambia	Kaoma	14° 48’00”S<br>24° 48’00”E	July 2013
Tanzania	Muheza	4° 54'18.00" S<br>38° 55'22.80" E	May 2018
	Tulizamoyo	8.354497° S	May 2018
	Ikwambi	7.98244° S<br>36.82167° E	May 2018
Uganda	Tororo	0° 45'N, 34° 5'E	March 2014
DR-Congo	Kinshasa	4° 19’39”S, 15° 18’48”E	May 2015
Cameroon	Lagdo	9° 05'N, 13° 40’E	June 2010

Table S2: List of BAC clone and *CYP6P9a* primers used for PCRs and sequencing

Primer	Size(bp) Range	Reverse	Forward	Tm (C)
0 BAC	716	TCACACTAGCTGCCAAATCG	TGGTAGCTGCTTGAGGAGAAA	57
25 BAC	823	TCATCTCCTTGAGTGCATCG	GTGACGGATCTGGACCTTGT	57
63 BAC	719	ACCAACCGGTAAGTTTCGTG	ATTACGAACCGTCAGCCATC	57
95 BAC	755	CTCCTCCGTAACGTGATCGT	CCTCTTTACTGGCCACCGTA	57
120 BAC	711	AGTTCGAGCACCAGCTCAAG	TCTTCCGCCATTGTGTATCA	57
CYP6P9a	815	TCACAATTTTTCCACCTTCAAGTAATTACCCGC	ATCCCTAACTATTAAAAGGCAAT	62
CYP6P9aIN	-	ACTCAAACGCTTTGTTGCCG	GCTTGTTGCGTCAGAAGCTC	67

Table S3a: Gene flow and genetic differentiation patterns

CYP6P9a	Fst	Gst	Nst	Hst	Kst
CYP6P9a full length 1,139bp					
Moz_P - Moz_M	0.00 (-)	-0.01 (-32.5)	0.00 (1*10E4)	-0.01	0.00
TZ_MU - TZ_TULI	0.02 (13.41)	-0.01 (-33.5)	0.02 (13.35)	-0.01	0.01
TZ_MU - TZ_IKW	0.00 (-2*1E15)	-0.01 (-33.5)	0.00 (2*10E4)	-0.01	0.00
TZ_TULI - TZ_IKW	0.03 (8.85)	0.00 (191.2)	0.03 (8.85)	-0.00	0.01
Southern Africa	0.02 (10.87)	0.04 (5.86)	0.02 (10.87)	0.05**	0.01***
Central Africa	0.53 (0.22)	0.25 (0.76)	0.53 (0.22)	0.25***	0.37***
South-East	0.97 (0.01)	0.28 (0.65)	0.97 (0.01)	0.28***	0.92***
South-Central	0.91 (0.02)	0.30 (0.57)	0.91 (0.02)	0.31***	0.87***
Central-East	0.84 (0.05)	0.15 (1.37)	0.84 (0.05)	0.15***	0.63***
ALL	0.93 (0.02)	0.44 (0.32)	0.93 (0.02)	0.44***	0.93***
CYP6P9a Coding Region 642 bp					
Moz_P - Moz_M	0.00 (-)	-0.01 (-32.5)	0.00 (6*10E3)	-0.01	0.00
TZ_MU - TZ_TULI	0.00 (-)	0.02 (-11.5)	-0.00 (2*10E3)	0.05	0.00
TZ_MU - TZ_IKW	0.00 (-2*1E15)	-0.01 (-31.6)	0.00 (1*10E4)	-0.01	0.00
TZ_TULI - TZ_IKW	0.00 (-)	0.00 (504.0)	0.00 (-)	0.02	0.00
Southern Africa	0.02 (14.52)	0.01 (19.94)	0.02 (14.53)	0.01	0.01*
Central Africa	0.68 (0.12)	0.42 (0.35)	0.68 (0.12)	0.43***	0.54***
South-East	1.0 (0.00)	0.61 (0.16)	1.0 (0.00)	0.61***	0.98***
South-Central	0.91 (0.02)	0.46 (0.30)	0.91 (0.02)	0.46***	0.88***
Central-East	0.87 (0.04)	0.35 (0.46)	0.87 (0.04)	0.34***	0.65***
ALL	0.96 (0.01)	0.69 (0.11)	0.96 (0.01)	0.69***	0.95***
CYP6P9a Non-Coding Region 497 bp					
Moz_P - Moz_M	-	-	-	-	-
TZ_MU - TZ_TULI	0.03 (6.94)	0.03 (9.74)	0.03 (6.96)	0.01	0.01
TZ_MU - TZ_IKW	-	-	-	-	-
TZ_TULI - TZ_IKW	0.03 (6.94)	0.03 (9.30)	0.03 (6.96)	0.01	0.02
Southern Africa	0.03 (9.59)	0.04 (6.33)	0.03 (9.59)	0.06**	0.02**
Central Africa	0.02 (10.5)	-0.01 (-28.2)	0.02 (10.39)	-0.01	0.01
South-East	0.88 (0.03)	0.40 (0.39)	0.89 (0.03)	0.40***	0.77***
South-Central	0.92 (0.02)	0.53 (0.22)	0.92 (0.02)	0.53***	0.87***
Central-East	0.78 (0.07)	0.29 (0.61)	0.78 (0.07)	0.29***	0.58***

Table S3b: Gene flow and genetic differentiation patterns

CYP6P9a	Fst	Gst	Nst	Hst	Kst
**CYP6P9a full length 1,139bp**					
Southern Africa	0.02 (10.87)	0.04 (5.86)	0.02 (10.87)	0.05**	0.01***
Central Africa	0.53 (0.22)	0.25 (0.76)	0.53 (0.22)	0.25***	0.37***
South-East	0.97 (0.01)	0.28 (0.65)	0.97 (0.01)	0.28***	0.92***
South-Central	0.91 (0.02)	0.30 (0.57)	0.91 (0.02)	0.31***	0.87***
Central-East	0.84 (0.05)	0.15 (1.37)	0.84 (0.05)	0.15***	0.63***
ALL	0.93 (0.02)	0.44 (0.32)	0.93 (0.02)	0.44***	0.93***
**CYP6P9a Coding Region 642 bp**					
Southern Africa	0.02 (14.52)	0.01 (19.94)	0.02 (14.53)	0.01	0.01*
Central Africa	0.68 (0.12)	0.42 (0.35)	0.68 (0.12)	0.43***	0.54***
South-East	1.0 (0.00)	0.61 (0.16)	1.0 (0.00)	0.61***	0.98***
South-Central	0.91 (0.02)	0.46 (0.30)	0.91 (0.02)	0.46***	0.88***
Central-East	0.87 (0.04)	0.35 (0.46)	0.87 (0.04)	0.34***	0.65***
ALL	0.96 (0.01)	0.69 (0.11)	0.96 (0.01)	0.69***	0.95***
**CYP6P9a Non-Coding Region 497 bp**					
Southern Africa	0.03 (9.59)	0.04 (6.33)	0.03 (9.59)	0.06**	0.02**
Central Africa	0.02 (10.5)	-0.01 (-28.2)	0.02 (10.39)	-0.01	0.01
South-East	0.88 (0.03)	0.40 (0.39)	0.89 (0.03)	0.40***	0.77***
South-Central	0.92 (0.02)	0.53 (0.22)	0.92 (0.02)	0.53***	0.87***
Central-East	0.78 (0.07)	0.29 (0.61)	0.78 (0.07)	0.29***	0.58***
ALL	0.87 (0.04)	0.55 (0.20)	0.87 (0.04)	0.56***	0.87***

Table S4a: Pairwise Fst distance based on 6p9a sequences from different countries

	MOZ_P	MOZ_M	MOZ	MAL	ZB	TZ-MU	TZ-TULI	TZ-IKW	TZ	DRC	CAM	UG
**CYP6P9a_Full_1139 bp**												
MOZ_P	-											
MOZ_M	0.000	-										
MOZ	0.000	0.000	-									
MAL	0.019	0.018	0.018	-								
ZB	0.035	0.031	0.032	0.030	-							
TZ-MU	0.000	0.000	0.000	0.006	0.024	-						
TZ-TULI	0.028	0.024	0.025	0.022	0.039	0.018	-					
TZ-IKW	0.000	0.000	0.000	0.019	0.034	0.000	0.027	-				
TZ	0.006	0.005	0.005	0.013	0.029	0.000	0.000	0.000	-			
DRC	0.958	0.956	0.957	0.947	0.951	0.953	0.952	0.957	0.954	-		
CAM	0.934	0.932	0.933	0.924	0.927	0.929	0.929	0.934	0.930	0.537	-	
UG	0.977	0.975	0.976	0.966	0.970	0.972	0.972	0.977	0.973	0.894	0.877	-
**CYP6P9a_Coding_642 bp**												
MOZ_P	-											
MOZ_M	0.000	-										
MOZ	0.000	0.000	-									
MAL	0.027	0.022	0.024	-								
ZB	0.000	0.000	0.000	0.037	-							
TZ-MU	0.000	0.000	0.000	0.003	0.000	-						
TZ-TULI	0.000	0.000	0.000	0.037	0.000	0.000	-					
TZ-IKW	0.000	0.000	0.000	0.027	0.000	0.000	0.000	-				
TZ	0.000	0.000	0.000	0.019	0.000	0.000	0.000	0.000	-			
DRC	0.983	0.980	0.981	0.978	0.985	0.975	0.985	0.982	0.981	-		
CAM	0.926	0.924	0.925	0.921	0.928	0.919	0.928	0.926	0.925	0.678	-	
UG	0.998	0.996	0.997	0.994	1.000	0.992	1.000	0.997	0.997	0.973	0.905	-
**CYP6P9a_Non-Coding_497 bp**												
MOZ_P	-											
MOZ_M	0.000	-										
MOZ	0.000	0.000	-									
MAL	0.012	0.012	0.012	-								
ZB	0.042	0.042	0.042	0.028	-							
TZ-MU	0.000	0.000	0.000	0.012	0.042	-						
TZ-TULI	0.035	0.035	0.035	0.016	0.039	0.035	-					
TZ-IKW	0.000	0.000	0.000	0.012	0.042	0.000	0.035	-				
TZ	0.014	0.014	0.014	0.009	0.035	0.014	0.000	0.014	-			
DRC	0.912	0.912	0.912	0.893	0.891	0.912	0.894	0.912	0.904	-		
CAM	0.950	0.950	0.950	0.928	0.925	0.950	0.929	0.950	0.941	0.023	-	
UG	0.899	0.899	0.899	0.869	0.865	0.899	0.870	0.899	0.887	0.766	0.806	-

Table S4b: Pairwise Fst distance based on 6p9a sequences from different countries

	Mozambique	Malawi	Zambia	Tanzania	Uganda	DR_Congo	Cameroon
**CYP6P9a_Full_1139 bp**							
**Mozambique**	-						
**Malawi**	0.018**	-					
**Zambia**	0.032	0.030	-				
**Tanzania**	0.005	0.013**	0.029	-			
**Uganda**	0.957***	0.947***	0.951***	0.954***	-		
**DR_Congo**	0.933***	0.924***	0.927***	0.930***	0.537***	-	
**Cameroon**	0.976***	0.966***	0.970***	0.973***	0.894***	0.877***	-
**CYP6P9a_Coding_642 bp**							
**Mozambique**	-						
**Malawi**	0.024	-					
**Zambia**	0.000	0.037	-				
**Tanzania**	0.000	0.019*	0.000	-			
**Uganda**	0.981***	0.978***	0.985***	0.981***	-		
**DR_Congo**	0.925***	0.921***	0.928***	0.925***	0.678***	-	
**Cameroon**	0.997***	0.994***	1.000***	0.997***	0.973***	0.905***	-
**CYP6P9a_Non-Coding_497 bp**							
**Mozambique**	-						
**Malawi**	0.012***	-					
**Zambia**	0.042*	0.028	-				
**Tanzania**	0.014	0.009*	0.035	-			
**Uganda**	0.912***	0.893***	0.891***	0.904***	-		
**DR_Congo**	0.950***	0.928***	0.925***	0.941***	0.023	-	
**Cameroon**	0.899***	0.869***	0.865***	0.887***	0.766***	0.806***	-

Table S5: Ratio of nucleotide diversity at *CYP6P9a* and five loci around the pyrethroid resistance gene.

	0BAC (-34kb)	25BAC (-9kb)	6P9A (0kb)	63BAC (+29kb)	95BAC (+61kb)	120BAC (+86kb)
Eastern / southern Africa	3.17117	0.00000	2.91667	0.75342	1.17822	1.55472
Central / southern Africa	6.51351	3.23967	7.00000	1.36301	2.42079	1.17736
East-Central / Southern Africa	4.84234	1.61983	1.40607	1.05822	1.79950	1.36604

**Table S6: Location of different BAC fragments regarding *An. funestus* genome annotation features from v

Chromosome	Start Position (Kb)	End Position (Kb)	Strand	Gene attribute
CM012071.1	8449150	8579233	+	AFUN020405 (solute carrier family 8; sodium/calcium exchanger)
**0BAC (-34kb)**	**8510505**	**8511221**	**+**	**within AFUN020405 locus**
CM012071.1	8531151	8533251	-	AFUN015786 (CYP6PAA1; cytochrome P450)
CM012071.1	8533523	8535535	-	AFUN015785 (CYP6PAA2; cytochrome P450)
**25BAC (-9kb)**	**8535505**	**8536328**	**-**	**within CYP6PAA2 and AFUN015785 loci**
CM012071.1	8535644	8537599	-	AFUN015787
CM012071.1	8539121	8540985	-	AFUN015793 (Carboxylic ester hydrolase)
CM012071.1	8541428	8543101	-	AFUN008357 (Cytochrome P450)
**CM012071.1**	**8544505**	**8546181**	**-**	**AFUN015792 (CYP6P9A; cytochrome P450)**
CM012071.1	8554228	8555956	-	AFUN015889 (CYP6P9b; cytochrome P450)
CM012071.1	8556748	8558550	-	AFUN015888 (CYP6P5; cytochrome P450)
CM012071.1	8559923	8562096	-	AFUN020895 (cytochrome P450)
CM012071.1	8562645	8564463	-	AFUN019365 (cytochrome P450)
CM012071.1	8565228	8566912	-	AFUN015802 (CYP6P1; cytochrome P450)
CM012071.1	8567086	8569039	-	AFUN015801 (CYP6P2; cytochrome P450)
CM012071.1	8569231	8571137	-	AFUN015714 (CYP6AD1; cytochrome P450)
**63BAC (+29kb)**	**8573505**	**8574224**	**-**	**Close to AFUN008362 locus**
CM012071.1	8582748	8584347	-	AFUN008362
CM012071.1	8586822	8589035	+	AFUN008363
CM012071.1	8597859	8599357	-	AFUN021910 (lipase)
**95BAC (+61kb)**	**8605505**	**8606260**	**-**	**Within AFUN021912 locus**
CM012071.1	8602367	8608947	-	AFUN021912
CM012071.1	8609970	8613744	-	AFUN021911
**120BAC (+86kb)**	**8630505**	**8631216**	**+**	**within AFUN008365 locus**

Table S7: Gene flow and genetic differentiation patterns

	Fst (Nm)	Gst (Nm)	Nst (Nm)	Hst	Kst
**0BAC**					
**Southern Africa**	0.02 (15.85)	0.02 (15.10)	0.02 (15.87)	0.01	0.01
**Central Africa**	0.76 (0.08)	0.34 (0.49)	0.76 (0.08)	0.36***	0.65***
**South-Central**	0.77 (0.07)	0.34 (0.49)	0.77 (0.07)	0.34***	0.68***
**South-East**	0.93 (0.02)	0.38 (0.41)	0.93 (0.02)	0.38***	0.83***
**East-Central**	0.51 (0.24)	0.32 (0.53)	0.51 (0.24)	0.32***	0.32***
**ALL**	0.87 (0.04)	0.51 (0.24)	0.87 (0.04)	0.51***	0.86***
**25BAC**					
**Southern Africa**	0.004 (56.20)	0.01 (26.32)	0.004 (55.94)	0.02	0.002
**Central Africa**	0.80 (0.06)	0.37 (0.42)	0.80 (0.06)	0.40***	0.72***
**South-Central**	0.67 (0.12)	0.45 (0.31)	0.67 (0.12)	0.45***	0.57***
**South-East**	0.98 (0.01)	0.66 (0.13)	0.98 (0.01)	0.66***	0.88***
**East-Central**	0.75 (0.08)	0.48 (0.27)	0.75 (0.08)	0.48***	0.55***
**ALL**	0.87 (0.04)	0.69 (0.11)	0.87 (0.04)	0.70***	0.88***
**63BAC**					
**Southern Africa**	0.00 (00)	0.006 (40.0)	0.00 (00)	0.007	0.00
**South-Central**	0.93 (0.02)	0.17 (1.26)	0.93 (0.02)	0.15***	0.73***
**South-East**	0.92 (0.02)	0.19 (1.08)	0.92 (0.02)	0.18***	0.75***
**East-Central**	0.92 (0.02)	0.28 (0.64)	0.92 (0.02)	0.28***	0.85***
**ALL**	0.91 (0.03)	0.26 (0.72)	0.91 (0.03)	0.26***	0.82***
**95BAC**					
**Southern Africa**	0.02 (11.82)	0.03 (9.62)	0.02 (11.82)	0.03*	0.02*
**Central Africa**	0.92 (0.02)	0.32 (0.54)	0.92 (0.02)	0.40***	0.88***
**South-Central**	0.62 (0.15)	0.27 (0.66)	0.62 (0.15)	0.28***	0.53***
**South-East**	0.93 (0.02)	0.36 (0.45)	0.92 (0.02)	0.36***	0.77***
**East-Central**	0.50 (0.25)	0.34 (0.48)	0.50 (0.25)	0.34***	0.33***
**ALL**	0.91 (0.02)	0.46 (0.29)	0.91 (0.02)	0.47***	0.87***
**120BAC**					
**Southern Africa**	0.03 (8.54)	0.02 (10.64)	0.03 (8.57)	0.03*	0.02**
**Central Africa**	0.91 (0.02)	0.71 (0.10)	0.91 (0.02)	0.76***	0.89***
**South-Central**	0.19 (1.08)	0.05 (4.37)	0.19 (1.08)	0.06***	0.12***
**South-East**	0.94 (0.01)	0.57 (0.19)	0.94 (0.01)	0.58***	0.87***
**East-Central**	0.84 (0.05)	0.42 (0.34)	0.84 (0.05)	0.42***	0.73***
**ALL**	0.88 (0.03)	0.60 (0.16)	0.88 (0.03)	0.61***	0.88***

*, 0.01<P<0.05; **, 0.001<P<0.01; ***, P<0.001

Table S8: Pairwise Fst distance between different countries

0BAC

	Mozambique	Malawi	Tanzania	Zambia	Uganda	DR_Congo	Cameroon
Mozambique	-						
Malawi	0.031*	-					
Tanzania	0.019*	0.017	-				
Zambia	0.001	0.021	0.002	-			
Uganda	0.949***	0.932***	0.920***	0.941***	-		
DR_Congo	0.977***	0.959***	0.947***	0.968***	0.874***	-	
Cameroon	0.849***	0.836***	0.825***	0.843***	0.601***	0.759***	-

25BAC

	Mozambique	Malawi	Tanzania	Zambia	Uganda	DR_Congo	Cameroon
Mozambique	-						
Malawi	0.018	-					
Tanzania	0.008	0.000	-				
Zambia	0.004	0.025	0.000	-			
Uganda	0.995***	0.967***	0.965***	0.972***	-		
DR_Congo	0.979***	0.943***	0.943***	0.951***	0.989***	-	
Cameroon	0.740***	0.706***	0.704***	0.717***	0.780***	0.780***	-

63BAC

	Mozambique	Tanzania	Uganda	DR_Congo
Mozambique	-			
Tanzania	0.000	-		
Uganda	0.932***	0.913***	-	
DR_Congo	0.927***	0.913***	0.916***	-

95BAC

	Mozambique	Malawi	Tanzania	Zambia	Uganda	DR_Congo	Cameroon
Mozambique	-						
Malawi	0.115	-					
Tanzania	0.036*	0.024	-				
Zambia	0.000	0.049*	0.000*	-			
Uganda	0.896***	0.946***	0.924***	0.873***	-		
DR_Congo	0.937***	0.970***	0.956***	0.921***	0.906***	-	
Cameroon	0.920***	0.935***	0.928***	0.913**	0.923***	0.925***	-

120BAC

	Mozambique	Malawi	Tanzania	Zambia	Uganda	DR_Congo	Cameroon
Mozambique	-						
Malawi	0.029	-					
Tanzania	0.019	0.010	-				
Zambia	0.030	0.03*	0.027**	-			
Uganda	0.956***	0.959***	0.955***	0.860***	-		
DR_Congo	0.029	0.000	0.010	0.030**	0.959***	-	
Cameroon	0.906***	0.911***	0.906***	0.797***	0.936***	0.911***	-
